# To develop a prognostic model for neoadjuvant immunochemotherapy efficacy in esophageal squamous cell carcinoma by analyzing the immune microenvironment

**DOI:** 10.3389/fimmu.2024.1312380

**Published:** 2024-04-25

**Authors:** Zhou Yehan, Qin Sheng, Yang Hong, Li Jiayu, Hou Jun, Ji Juan, Shi Min, Yan Jiaxin, Hu Shangzhi, Wang Yi, Wang Qifeng, Leng Xuefeng, He Wenwu, Cheng Xueyan, Liu Yang, Huang Zongyao

**Affiliations:** ^1^ Department of Pathology, Sichuan Cancer Hospital & Institute, Sichuan Cancer Center, School of Medicine, University of Electronic Science and Technology of China, Chengdu, China; ^2^ Department of Endoscopy Center, Sichuan Cancer Hospital & Institute, Sichuan Cancer Center, School of Medicine, University of Electronic Science and Technology of China, Chengdu, China; ^3^ Department of Radiotherapy, Sichuan Cancer Hospital & Institute, Sichuan Cancer Center, School of Medicine, University of Electronic Science and Technology of China, Chengdu, China; ^4^ Department of Thoracic Surgery, Sichuan Cancer Hospital & Institute, Sichuan Cancer Center, School of Medicine, University of Electronic Science and Technology of China, Chengdu, China; ^5^ Genecast Biotechnology Co., Ltd, Wuxi, China

**Keywords:** esophageal squamous cell carcinoma (ESCC), neoadjuvant immunochemotherapy (NICT), response, tumor immune microenvironment (TIME), predictive model

## Abstract

**Objective:**

The choice of neoadjuvant therapy for esophageal squamous cell carcinoma (ESCC) is controversial. This study aims to provide a basis for clinical treatment selection by establishing a predictive model for the efficacy of neoadjuvant immunochemotherapy (NICT).

**Methods:**

A retrospective analysis of 30 patients was conducted, divided into Response and Non-response groups based on whether they achieved major pathological remission (MPR). Differences in genes and immune microenvironment between the two groups were analyzed through next-generation sequencing (NGS) and multiplex immunofluorescence (mIF). Variables most closely related to therapeutic efficacy were selected through LASSO regression and ROC curves to establish a predictive model. An additional 48 patients were prospectively collected as a validation set to verify the model’s effectiveness.

**Results:**

NGS suggested seven differential genes (ATM, ATR, BIVM-ERCC5, MAP3K1, PRG, RBM10, and TSHR) between the two groups (P < 0.05). mIF indicated significant differences in the quantity and location of CD3+, PD-L1+, CD3+PD-L1+, CD4+PD-1+, CD4+LAG-3+, CD8+LAG-3+, LAG-3+ between the two groups before treatment (P < 0.05). Dynamic mIF analysis also indicated that CD3+, CD8+, and CD20+ all increased after treatment in both groups, with a more significant increase in CD8+ and CD20+ in the Response group (P < 0.05), and a more significant decrease in PD-L1+ (P < 0.05). The three variables most closely related to therapeutic efficacy were selected through LASSO regression and ROC curves: Tumor area PD-L1+ (AUC= 0.881), CD3+PD-L1+ (AUC= 0.833), and CD3+ (AUC= 0.826), and a predictive model was established. The model showed high performance in both the training set (AUC= 0.938) and the validation set (AUC= 0.832). Compared to the traditional CPS scoring criteria, the model showed significant improvements in accuracy (83.3% vs 70.8%), sensitivity (0.625 vs 0.312), and specificity (0.937 vs 0.906).

**Conclusion:**

NICT treatment may exert anti-tumor effects by enriching immune cells and activating exhausted T cells. Tumor area CD3+, PD-L1+, and CD3+PD-L1+ are closely related to therapeutic efficacy. The model containing these three variables can accurately predict treatment outcomes, providing a reliable basis for the selection of neoadjuvant treatment plans.

## 1 Introduction

China accounts for about half of the world’s esophageal cancer cases, predominantly squamous cell carcinoma. Unfortunately, most of these cases are identified at an advanced stage (1). Combining neoadjuvant therapy with surgery has shown benefits for these patients. The adoption of neoadjuvant chemoradiotherapy as a standard preoperative treatment has been influenced by the results of the CROSS and 5010 studies (2, 3). Recently, the advent of immunotherapy, highlighted by the CheckMate-648 and Keynote-590 studies, has introduced neoadjuvant immunochemotherapy (NICT) as an effective alternative, demonstrating comparable rates of pathological remission to neoadjuvant chemoradiation therapy (NCRT) without increasing surgical complexity or side effects, positioning NICT as a potentially superior option (4, 5). Given this context, accurately predicting NICT’s effectiveness is crucial, with major pathological remission (MPR) being a key metric for assessing NICT’s success. Accurate MPR prediction is vital for selecting appropriate neoadjuvant treatments (6).

Research has explored various biomarkers, such as PD-L1 score, tumor mutation burden (TMB), tumor-infiltrating lymphocytes (TILs), and microsatellite instability (MSI), to predict immunotherapy outcomes in solid tumors like those in the esophagus, breast, and intestines (7–9). However, inconsistent and sometimes contradictory clinical trial results indicate significant variability in individual and tumor-specific responses to immunotherapy, likely due to the tumor immune microenvironment’s complexity.

The tumor’s immune microenvironment, shaped by the quantity, distribution, and function of immune cells, plays a crucial role in the anti-tumor response. Studies, including the CLEOPATRA study, suggest that a high presence of TILs can enhance the immune system’s ability to attack cancer cells (10–12). The spatial arrangement of immune cells, as observed in lung cancer, and the interaction among different types of immune cells can significantly influence the treatment outcome.

Recent advancements, such as multiplex immunofluorescence technology (mIF), combined with machine learning, offer new ways to analyze the immune microenvironment by quantifying and characterizing immune cells. This approach aims to identify key features that can predict the effectiveness of neoadjuvant treatments, thereby aiding clinical decision-making.

## 2 Study subjects and methods

### 2.1 Subjects

The research objects were derived from the surgical resection specimens of esophageal squamous cell carcinoma admitted to Sichuan Cancer Hospital from December 2020 to December 2022. Inclusion criteria: (1) primary advanced esophageal squamous cell carcinoma, (2) no other malignant tumors, (3) NICT treatment before surgery, (4) radical resection of esophageal cancer after NICT treatment, Exclusion criteria (1) with immunodeficiency related diseases.

**Figure 1:**
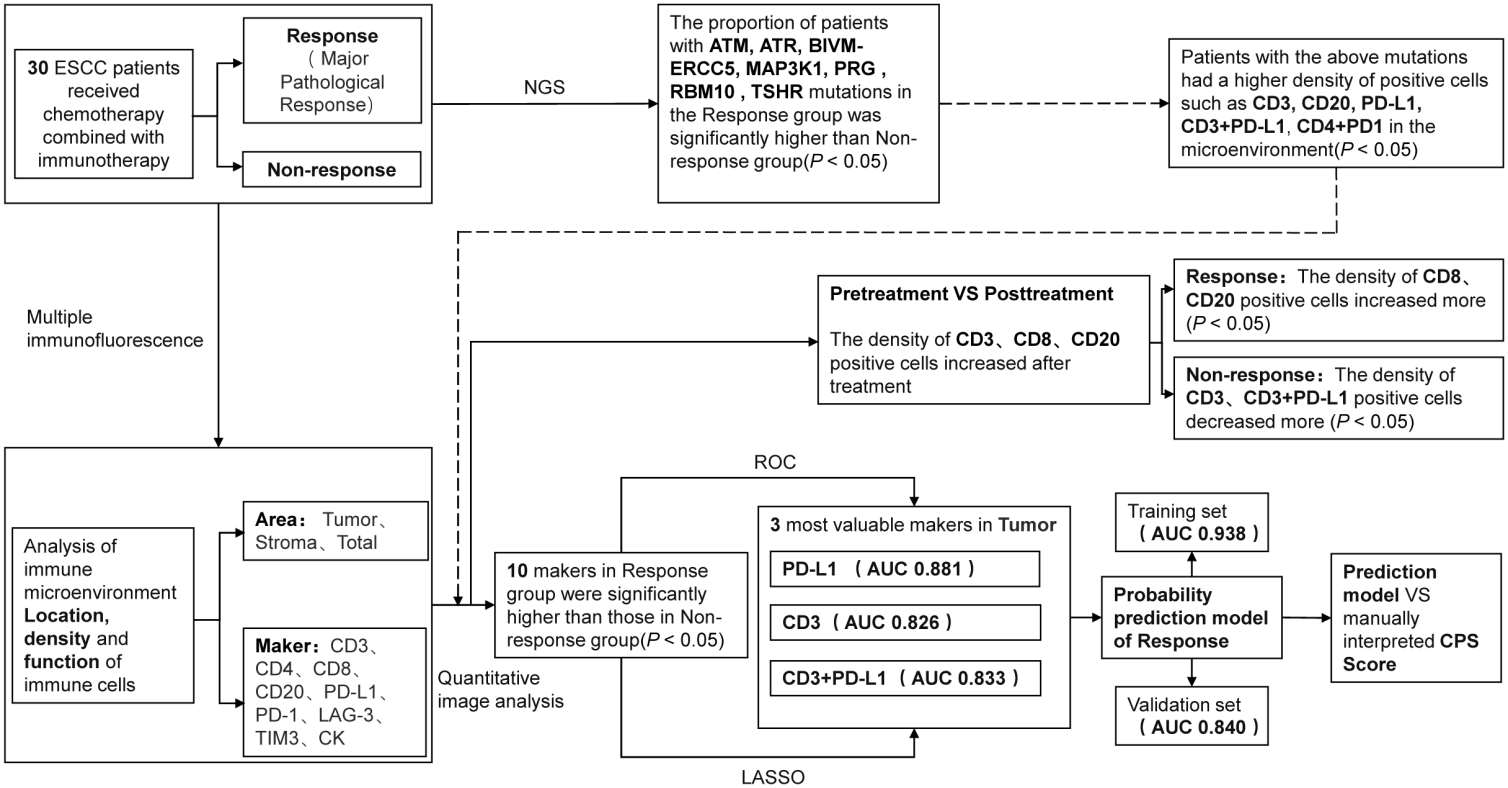
The flow chart of the study.

A total of 78 patients were enrolled in the study. Patients were treated with neoadjuvant chemotherapy combined with immunotherapy before surgery. Albumin paclitaxel, carboplatin, cisplatin, docetaxel, oxaliplatin and other single or double drugs were used for chemotherapy, and Nivolumab, Tislelizumab, Pembrolizumab and Sintilizumab were used for immunotherapy. All patients received 1-4 cycles of treatment, with an average of 2.13 ± 0.58 cycles. Detailed experimental procedures can be found in the research roadmap (**Figure 1**).

### 2.2 Methods

#### 2.2.1 Sample treatment

The study involved detailed tissue processing and analysis techniques. Specimens were preserved in 10% neutral buffered formalin and then encased in paraffin. Following standard procedures, these were then sectioned and subjected to hematoxylin and eosin (H&E) staining for basic histological examination.

For immunohistochemistry (IHC), the Streptavidin-peroxidase (SP) method was employed, utilizing an automatic IHC instrument to ensure precision and consistency. This method involved a series of carefully controlled staining steps as per the instrument’s guidelines.

Multiplex immunofluorescence (mIF) staining was outsourced to Genecast Biotechnology Co., Ltd. This advanced technique was applied to detect a comprehensive set of 10 biomarkers, divided into two panels. Panel 1 included markers CD4, CD8, PD-1, TIM3, and LAG-3, while Panel 2 comprised CD3, CD20, CD21, PD-L1, and PCK. These markers are crucial for identifying various immune cells and their states, with CD3, CD20, and CD21 specifically highlighting tertiary lymphoid structures (TLS).The process for each panel began with preparing 4-μm thick sections from formalin-fixed paraffin-embedded (FFPE) esophageal squamous cell carcinoma (ESCC) tissues. These sections were then deparaffinized, rehydrated, and subjected to epitope retrieval by boiling in Tris-EDTA buffer. To prevent nonspecific binding, endogenous peroxidase activity was blocked, followed by protein blockage in a Tween solution. The antigens in each panel were detected through cyclic staining, which involves the sequential application of primary and secondary antibodies, visualization using tyramine signal amplification (TSA), and the removal of the TSA-antibody complex. This cycle was repeated for each marker, with epitope retrieval and protein blocking conducted in each round to ensure specificity and clarity of staining. Finally, each slide was counterstained with DAPI to mark the cell nuclei and mounted with an antifade mountant to preserve the fluorescence, facilitating detailed microscopic examination and analysis of the tissue samples.

#### 2.2.2 Quantitative image analysis

The imaging and analysis of the tissue samples were performed using the TissueFAXS panoramic tissue cell imaging quantitative analysis system (TissueFAXS SL Plus S, Austria, Tissue Gnostics). The process began with a low-magnification (2.5X) preview of the entire slide to locate the tissue sections. Optimal imaging conditions, including exposure time and value, were then adjusted based on the expression of targets in each dye channel, ensuring the best quality scans. The areas of interest were selected from the preview and scanned at a 20X magnification under these conditions.

For the analysis, Strata Quest software (version 7.1.129, Austria, Tissue Gnostics) was utilized to segment and identify cells and tissue types within the scanned images. This software employs intelligent algorithms to segment cells based on nuclear staining and integrates manual training with machine learning for accurate tissue type classification into categories like tumor and stroma. Protein expression levels were quantified by setting average fluorescence thresholds for each marker, with cells exceeding these thresholds considered positive for the respective markers.

#### 2.2.3 Genetic testing technique

In addition to imaging, genetic testing techniques were applied to the study. DNA extracted from formalin-fixed paraffin-embedded (FFPE) samples was fragmented and prepared into libraries using the KAPA Hyper Preparation Kit. These libraries were then assessed and quantified, with targeted regions selected for further analysis. Hybridization and washing protocols were followed as per the manufacturer’s guidelines, with sequencing conducted on the Illumina Novaseq 6000 system. The sequencing data were processed using established bioinformatics tools for alignment, sorting, and variant calling.

#### 2.2.4 Statistical analysis

was conducted using logistic regression, Wilcoxon test, and the least absolute shrinkage and selection operator (LASSO) regression to analyze the data. The study utilized a split-sample approach, with thirty retrospective samples forming the training set and forty-eight prospective samples serving as the validation set. The model’s performance was evaluated using receiver operating characteristic (ROC) curves. All statistical analyses were carried out using R statistical software and the FreeStatistics platform, with a p-value of less than 0.05 considered indicative of statistical significance.

## 3 Results

### 3.1 Pathological and clinical characteristics

**Figure 2:**
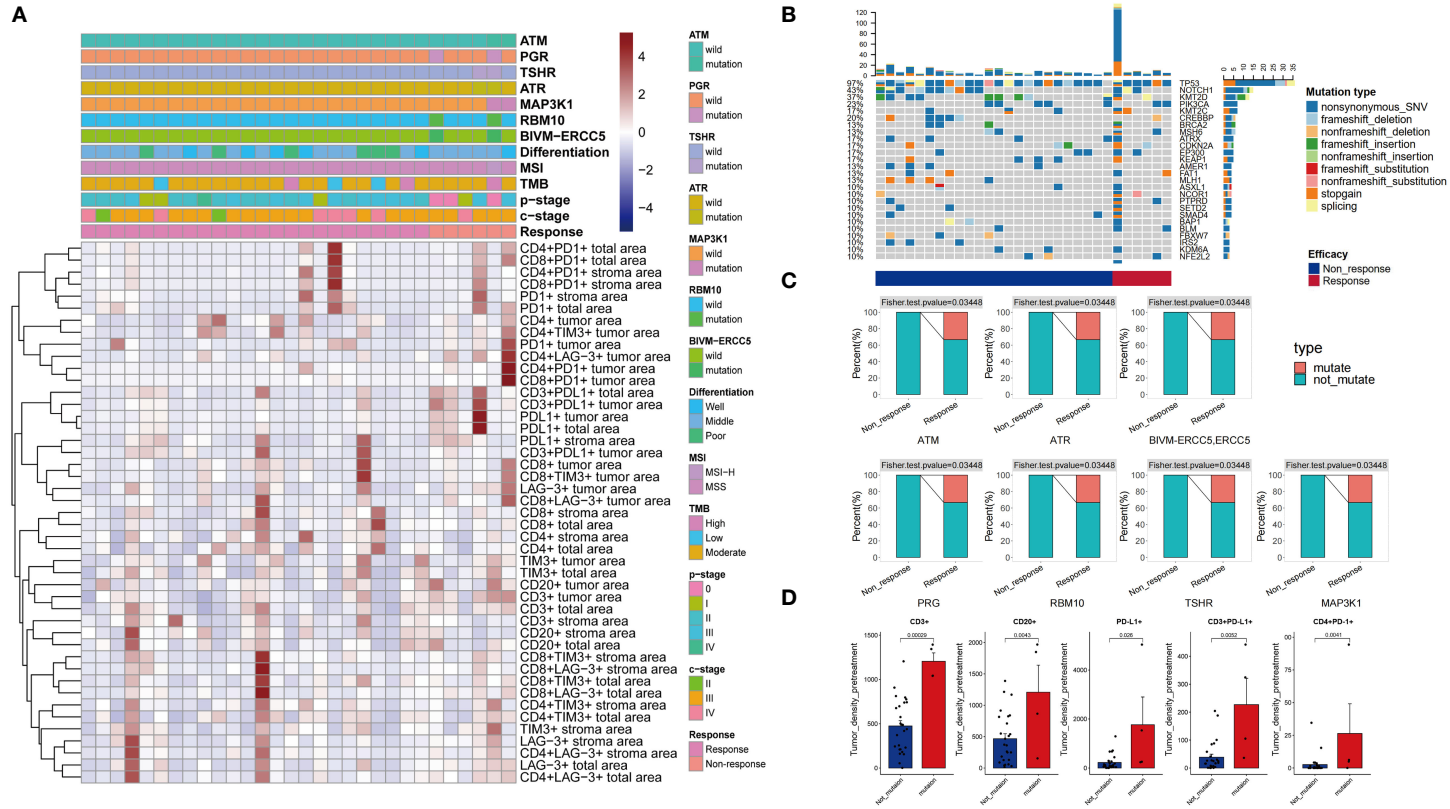
Clinical and pathological information of patients **(A)**. Patient mutation information **(B)**. Differential mutations were detected in Response and Non-response groups **(C)**. Differences in immune microenvironment between Mutate and Not-mutate groups **(D)**.

The 30 patients were aged 59.00 [55.25, 65.75] years, with 27 (90%) being male and 28 (93.3%) at clinical stages III-IV. They received 2.17 ± 0.58 cycles of chemotherapy combined with immunotherapy, with 6 (20%) achieving Response (MPR) (**Figure 2A**). There were no statistically significant differences in gender, age, medical history, stage, differentiation, and location between the Response and Non-response groups (**Table 1**).

**Table 1:** Clinicopathological characteristics.

Characteristic	Total (n = 30)	Response (n = 6)	Non-response (n = 24)	p
**Gender, n (%)**				1
**Female**	3 (10.0)	0 (0)	3 (12.5)	
**Male**	27 (90.0)	6 (100)	21 (87.5)	
**Age, n (%)**				0.3
**≤65**	22 (73.3)	3 (50)	19 (79.2)	
**>65**	8 (26.7)	3 (50)	5 (20.8)	
**Cigarette smoking history, n (%)**				1
**No**	7 (23.3)	1 (16.7)	6 (25)	
**Yes**	23 (76.7)	5 (83.3)	18 (75)	
**Alcohol drinking history, n (%)**				1
**No**	9 (30.0)	2 (33.3)	7 (29.2)	
**Yes**	21 (70.0)	4 (66.7)	17 (70.8)	
**Family history, n (%)**				1
**No**	25 (83.3)	5 (83.3)	20 (83.3)	
**Yes**	5 (16.7)	1 (16.7)	4 (16.7)	
**cT, n (%)**				1
**T2**	3 (10.0)	0 (0)	3 (12.5)	
**T3**	20 (66.7)	5 (83.3)	15 (62.5)	
**T4**	7 (23.3)	1 (16.7)	6 (25)	
**cN, n (%)**				0.766
**0**	2 (6.7)	0 (0)	2 (8.3)	
**1**	20 (66.7)	5 (83.3)	15 (62.5)	
**2**	8 (26.7)	1 (16.7)	7 (29.2)	
**stage, n (%)**				1
**II**	2 (6.7)	0 (0)	2 (8.3)	
**III**	21 (70.0)	5 (83.3)	16 (66.7)	
**IV**	7 (23.3)	1 (16.7)	6 (25)	
**Differentiation, n (%)**				0.574
**Well**	6 (20.0)	1 (16.7)	5 (20.8)	
**Middle**	18 (60.0)	5 (83.3)	13 (54.2)	
**Poor**	6 (20.0)	0 (0)	6 (25)	
**Primary tumor location, n (%)**				0.166
**Lower**	18 (60.0)	3 (50)	15 (62.5)	
**Middle**	9 (30.0)	1 (16.7)	8 (33.3)	
**Upper**	3 (10.0)	2 (33.3)	1 (4.2)	

### 3.2 Gene testing results

A total of 191 mutated genes were detected in the 30 patients, with the most frequently mutated genes being TP53 (97%), NOTCH1 (43%), KMT2D (37%), PIK3CA (23%), and CREBBP (20%) (**Figure 2B**). Three (10%) patients were TMB-H, and one (3.3%) patient was MSI-H. TMB-H, MSI-H was more present in the Response group, but it was not statistically significant. Seven genes, ATM, ATR, BIVM-ERCC5, MAP3K1, PRG, RBM10, and TSHR, had significantly higher mutation rates in the Response group than in the Non-response group (P < 0.05) (**Figure 2C**). The density of Tumor area PD-L1+, CD3+PD-L1+, CD3+, CD20+, CD4+PD-1+ cells was significantly higher in the population with the above seven gene mutations than in the population without these mutations (P < 0.05) (**Figure 2D**).

### 3.3 Multiplex immunofluorescence immune microenvironment test results

#### 3.3.1 Pre-treatment immune microenvironment analysis

**Figure 3:**
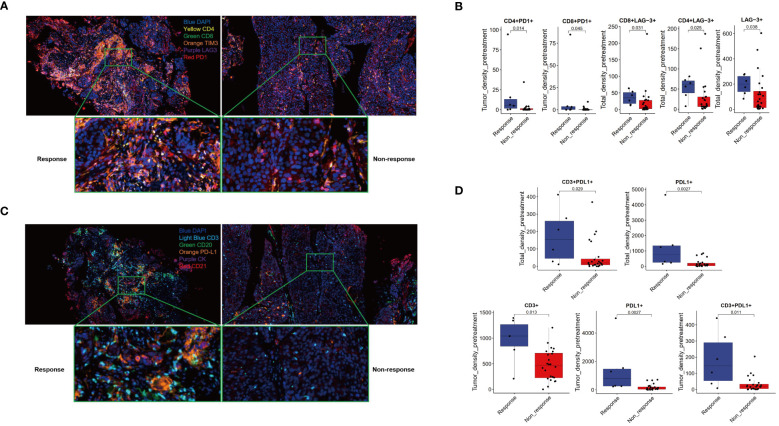
Multiple immunofluorescence technology was used to analyze the immune microenvironment. Panel 1.2 Schematic diagram **(A, C)** and image quantitative analysis results **(B, D)**.

The study utilized two panels of detection markers to analyze tumor immune environments. In Panel 1, the markers included CD4, CD8, PD-1, TIM3, LAG-3, and DAPI. The findings, illustrated in **Figure 3A**, revealed distinct immune responses within the tumor microenvironments. The Response group displayed dense clusters of CD4+ and CD8+ T cells within and surrounding the tumor, indicative of a highly active (“hotter”) immune response. Conversely, the Non-response group showed fewer and more scattered T cells, suggestive of a less active (“colder”) immune environment (**Figure 3A**). Quantitative analyses further confirmed these observations, showing significantly greater densities of CD4+PD-1+ and CD8+PD-1+ cells in the Response group compared to the Non-response group within the tumor region. Additionally, the Response group exhibited higher densities of various T cell markers, indicating a more robust immune presence (**Figure 3B**, **Supplementary Figure 1**).

Panel 2 focused on markers CD3, CD20, CD21, PD-L1, PCK, and DAPI, with an emphasis on identifying tertiary lymphoid structures (TLS). **Figure 3C** presents the comparative immune landscapes, where the Response group showed denser distributions of CD3+ T cells and higher PD-L1 expression in both T cells and tumor cells, suggesting potential TLS presence (**Figure 3C**). The Non-response group, however, displayed sparser CD3+ T cells and lower PD-L1 expression, with an absence of TLS. Quantitative data supported these visual findings, with the Response group showing significantly higher densities of CD3+, PD-L1+, and CD3+PD-L1+ cells within the tumor. Moreover, the Response group exhibited a trend toward higher densities of CK+PD-L1+ cells across all examined regions. While the TLS density was somewhat higher in the Response group, the difference was not statistically significant (**Figure 3D**, **Supplementary Figure 2**).

These analyses underscore the complex interplay within the tumor microenvironment and highlight the potential of immune markers in predicting treatment responses.

#### 3.3.2 Dynamic analysis of the immune microenvironment before and after treatment

**Figure 4:**
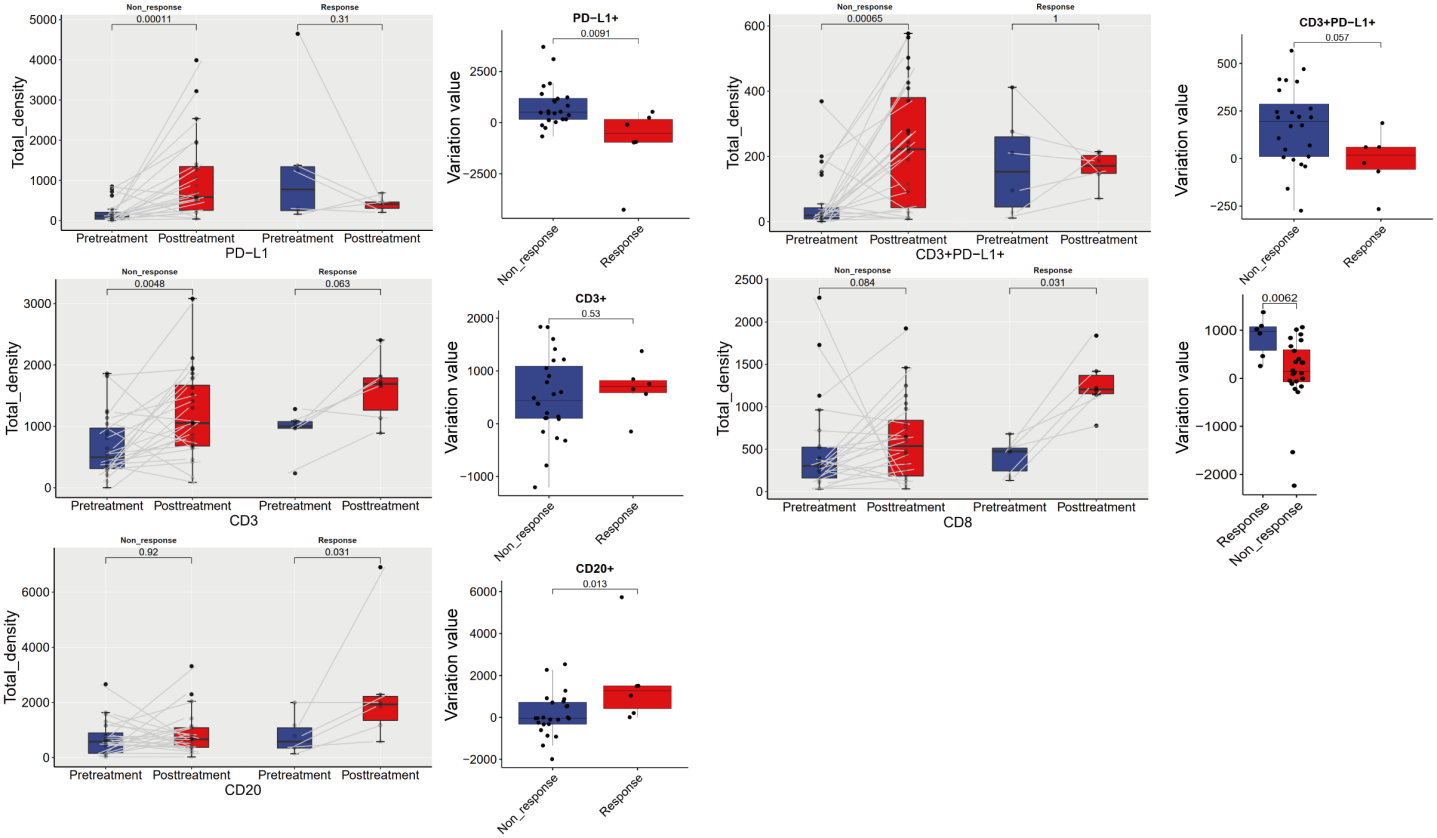
Dynamic analysis of immune microenvironment before and after treatment.

The densities of CD3+, CD8+, and CD20+ cells in both the Response and Non-response groups increased after treatment, with a more significant increase in CD8+ and CD20+ in the Response group (P < 0.05). The density of PD-L1+ cells significantly increased after treatment in the Non-response group (P < 0.05), while it decreased after treatment in the Response group, with a significant difference in the change values between the two groups (P < 0.05). The density of CD3+ PD-L1+ cells significantly increased after treatment in the Non-response group (P < 0.05), while the change was not significant after treatment in the Response group (**Figure 4**). The densities of PD-1+, TIM3+, LAG-3+, CD4+PD-1+, CD4+ LAG-3+, CD8+PD-1+, CD8+TIM3+, CD8+LAG-3+ cells showed a trend of being basically unchanged or decreased after treatment (**Supplementary Figure 3**), and the change values were not statistically significant (P > 0.05) (**Supplementary Figure 4**).

### 3.4 Establishment and verification of the prediction model

**Figure 5:**
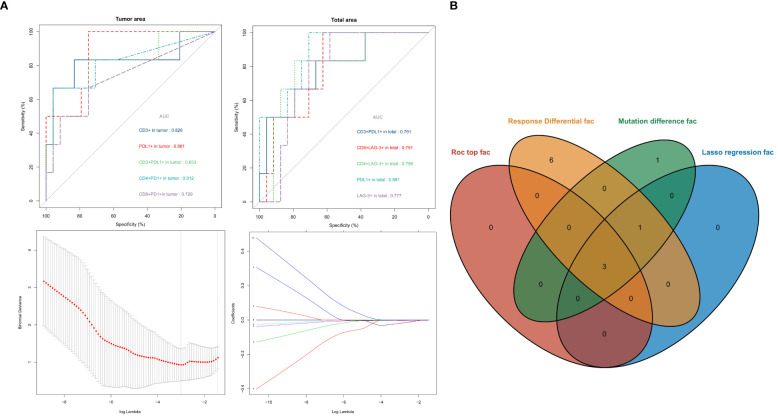
ROC curves for each variable [upper of **(A)**]. Lasso regression screened variables [lower of **(A)**]. Intersection of variables selected by multiple methods **(B)**.

**Figure 6:**
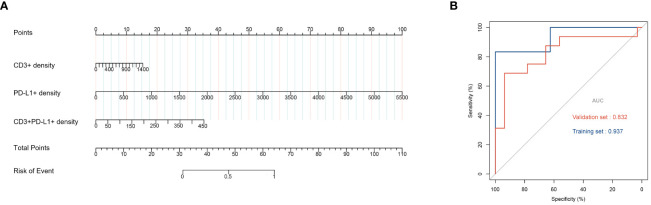
Nomogram of the prediction model **(A)**. ROC curve of training set and validation set **(B)**.

The 10 variables in Panel 1 and 2 with significant differences between the Non-response and Response groups were analyzed by ROC curve to evaluate their value in predicting the response to neoadjuvant therapy. The AUC values were ranked from high to low as follows: Tumor and Total area PD-L1+ (AUC= 0.881), Tumor area CD3+PD-L1+ (AUC= 0.833), Tumor area CD3+ (AUC= 0.826), Tumor area CD4+PD-1+ (AUC= 0.812), Total area CD4+LAG-3+ (AUC= 0.798), Total area CD8+LAG-3+ (AUC= 0.791), Total area CD3+PD-L1+ (AUC= 0.791), Total area LAG-3+ (AUC= 0.777), Tumor area CD8+PD-1 (AUC= 0.729). All variables were analyzed by LASSO regression, and when the λ coefficient decreased to the optimum as the number of variables increased (**Figure 3B**), four variables with non-zero coefficients were selected: Tumor area CD3+, PDL1+, CD3+PD-L1+, CD4+PD-1+ (**Figure 5A**). The intersection of the 10 variables with significant differences between the two groups, the top 3 variables in ROC, the 4 variables screened by LASSO regression, and the 5 variables with significant differences between the mutation and non-mutation groups were taken to make a Venn diagram (**Figure 5B**). Finally, three variables were selected to establish a model with high clinical accessibility, and a nomogram was obtained to predict the patient’s Response. The nomogram reflected the predictive value of CD3+, PD-L1+ and CD3+PD-L1+ for the occurrence of MPR. The scoring system was developed based on the densities of CD3+, PD-L1+, and CD3+PD-L1+ cells within the tissue samples. Specifically, the density of CD3+ cells ranged from 0 to 1400 cells per square millimeter, which was translated into a score ranging from 0 to 15 points. Similarly, the density of PD-L1+ cells spanned from 0 to 5500 cells per square millimeter, corresponding to a score range of 0 to 100 points. For the combined presence of CD3+ and PD-L1+ cells, densities from 0 to 450 cells per square millimeter were assigned scores from 0 to 35 points. Densities exceeding these maximum thresholds were allocated the highest possible score for each category. The aggregation of these three individual scores yielded a composite score, which was directly associated with the likelihood of the patient achieving a Major Pathological Response (MPR), as illustrated in (**Figure 6A**). The AUC value of the model in the training set was higher than that of a single variable, reaching 0.938 (**Figure 6B**).

**Figure 7:**
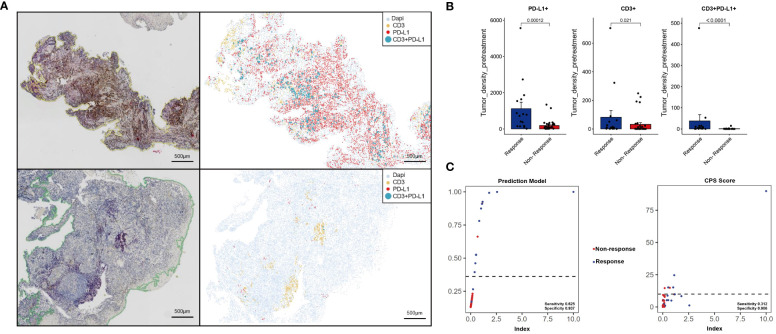
Immunohistochemistry and machine learning analysis were used to analyze the immune microenvironment of the validation set. Response [upper of **(A)**], Non-response [lower of **(A)**]. Image quantitative analysis results **(B)**. The predictive effects of the prediction model and CPS scoring system were compared **(C)**.

In the validation set, a more economical IHC method was used for CD3 (red) and PD-L1 (brown) double staining. IHC showed that Response patients had higher PD-L1 expression and more CD3+T cell aggregation (**Figure 7A** the upper left), and the AI data analysis platform showed that Response patients presented a “hotter” microenvironment simulation map (**Figure 7A** the upper right). And compared to the Non-response group, the Response group had significantly higher densities of PD-L1+, CD3, CD3+PD-L1+ cells (P < 0.05) (**Figure 7B**). The AUC value of the model in the validation set reached 0.832 (**Figure 6B**). Model scores (linear predictors) were significantly associated with treatment response (MPR) (OR=2.72, 95%CI 1.36-5.43, P = 0.005). Through the hosmer-lemeshow model fitting test, it was considered that there was no significant difference between the model prediction result and the actual result (P = 0.183). Compared with the traditional PD-L1 CPS score standard (13), the prediction model showed some improvement in accuracy (83.3% vs 70.8%), sensitivity (0.625 vs 0.312), and specificity (0.937 vs 0.906) (**Figure 7C**).

## 4 Discussion

Our study employed advanced multiplex immunofluorescence and quantitative imaging techniques to explore the immune landscape in esophageal squamous cell carcinoma (ESCC) patients prior to treatment. We discovered significant differences in the immune environments between patients who responded to neoadjuvant immunotherapy (NICT) and those who didn’t, particularly in terms of the presence and activity of immune cells. Key indicators for predicting NICT outcomes included the concentration of CD3+ T cells, CD3+PD-L1+ T cells, and overall PD-L1+ cells within the tumor. A predictive model based on these indicators showed promising accuracy in distinguishing between response outcomes, and importantly, it can be replicated using cost-effective immunohistochemistry, offering a practical tool for clinical predictions of NICT success.

Initially, our search for molecular markers linked to treatment responses involved extensive next-generation sequencing. Although high tumor mutational burden (TMB-H) and microsatellite instability (MSI-H) were more common in responders, the differences weren’t statistically significant. We also identified variations in several genes, including MAP3K1 (14) and ERCC5 (15), known to influence chemotherapy sensitivity, and ATM, ATR (16)and RBM10 (17), which are involved in DNA repair and tumor immunity. Immunogenic cell death (ICD) -related genes are of great significance for the prognosis and microenvironment regulation of lung adenocarcinoma (18). However, more research is needed for esophageal cancer. While these molecular markers hint at potential immunochemotherapy benefits, their predictive value for NICT efficacy in ESCC remains to be further explored.

Our research highlights the crucial role of immune cell quantity, distribution, and functionality within the tumor microenvironment. We found a higher count of various immune cells, such as B cells, helper, and cytotoxic T cells in responders, suggesting a robust immune response (19–21). Interestingly, the distribution of immune cells within the tumor, rather than the surrounding stroma, was particularly indicative of treatment efficacy. This aligns with previous findings where the presence of immune cells within tumors correlated with better outcomes in other cancers (22, 23).

Furthermore, the study sheds light on the functional aspects of immune cells, emphasizing the importance of CD20+ B cells and their role in forming tertiary lymphoid structures, which are beneficial for immunotherapy (24). The density of T cells, particularly CD3+ cells, was significantly higher in responders, underscoring their potential for predicting pathological remission (25–28). Of course, numerous immune checkpoint-related biomarkers are potentially valuable for efficacy evaluation (29–36).

The dynamic analysis of samples before and after NICT treatment revealed an increase in immune cell populations, especially in responders, reinforcing the link between immune cell accumulation and treatment success (37, 38). Interestingly, PD-L1 expression decreased in responders but increased in non-responders post-treatment, suggesting NICT may reinvigorate exhausted T cells, enhancing the immune response (39, 40).

There are many indicators related to prognosis (41–43). The immune characteristic risk model genophenotype predicts efficacy (44), and BMI-LMR scores and pan-immune-inflammation value (PIV) predict prognosis through systemic indicators (45, 46). This is one of the few studies to develop a predictive model by analyzing the immune microenvironment. This model is simple and inexpensive, and therefore has high clinical accessibility. Despite the promising results, our study acknowledges the need for broader validation and recognizes the limitations of relying solely on local microenvironment analysis. Combining systemic immune status indicators with local analysis could enhance predictive accuracy. Our model, while specific and less costly than some alternatives (45, 46), still requires further validation to confirm its applicability and reliability in clinical settings.

## Data availability statement

The data presented in the study are deposited in the Figshare (https://doi.org/10.6084/m9.figshare.23951592) repository.

## Ethics statement

The studies involving humans were approved by Ethical approval Permission has been obtained from the Ethics Committee for Medical Research and New Medical Technology of Sichuan Cancer Hospital (Nos: SCCHEC-02-2022-011). The studies were conducted in accordance with the local legislation and institutional requirements. The participants provided their written informed consent to participate in this study.

## Author contributions

ZY: Data curation, Methodology, Supervision, Validation, Writing – original draft, Writing – review & editing. QS: Data curation, Writing – review & editing. YH: Data curation, Writing – review & editing. LJ: Data curation, Writing – review & editing. HJ: Data curation, Writing – review & editing. JJ: Data curation, Writing – review & editing. SM: Data curation, Writing – review & editing. YJ: Data curation, Writing – review & editing. HS: Data curation, Writing – review & editing. WY: Data curation, Writing – review & editing. WQ: Data curation, Writing – review & editing. LX: Data curation, Writing – review & editing. HW: Data curation, Writing – review & editing. CX: Data curation, Writing – review & editing. LY: Project administration, Writing – review & editing. HZ: Data curation, Funding acquisition, Methodology, Project administration, Supervision, Validation, Writing – review & editing.

## Supplementary material

Supplementary Figure 1 Quantitative analysis results of panel 1.

Supplementary Figure 2 Quantitative analysis results of panel 2.

Supplementary Figure 3 The dynamic analysis results of the two groups before and after treatment.

Supplementary Figure 4 The variation value analysis results of the two groups before and after treatment.

Supplementary Figure 5 Model calibration curves in training set **(A)** and validation set **(B)**.

Supplementary Figure 6 Patient mutation information.

Supplementary Table 1 Clinicopathological characteristics of validation set.

## References

[1] Zhu H Ma X Ye T Wang H Wang Z Liu Q. Esophageal cancer in China: Practice and research in the new era. Int J Cancer. (2023) 152:1741–51. doi: 10.1002/ijc.34301 36151861

[2] van Hagen P Hulshof MC van Lanschot JJ Steyerberg EW van Berge Henegouwen MI Wijnhoven BPL. Preoperative chemoradiotherapy for esophageal or junctional cancer. New Engl J Med. (2012) 366:2074–84. doi: 10.1056/NEJMoa1112088 22646630

[3] Yang H Liu H Chen Y Zhu C Fang W Yu Z. Neoadjuvant chemoradiotherapy followed by surgery versus surgery alone for locally advanced squamous cell carcinoma of the esophagus (NEOCRTEC5010): A phase III multicenter, randomized, open-label clinical trial. J Clin Oncol. (2018) 36:2796–803. doi: 10.1200/JCO.2018.79.1483 PMC6145832 30089078

[4] Doki Y Ajani JA Kato K Xu J Wyrwicz L Motoyama S. Nivolumab combination therapy in advanced esophageal squamous-cell carcinoma. New Engl J Med. (2022) 386:449–62. doi: 10.1056/NEJMoa2111380 35108470

[5] Sun JM Shen L Shah MA Enzinger P Adenis A Doi T. Pembrolizumab plus chemotherapy versus chemotherapy alone for first-line treatment of advanced oesophageal cancer (KEYNOTE-590): a randomised, placebo-controlled, phase 3 study. Lancet. (2021) 398:759–71. doi: 10.1016/S0140-6736(21)01234-4 34454674

[6] Xu L Wei XF Li CJ. Pathologic responses and surgical outcomes after neoadjuvant immunochemotherapy versus neoadjuvant chemoradiotherapy in patients with locally advanced esophageal squamous cell carcinoma. Front Immunol. (2022) 13:1052542. doi: 10.3389/fimmu.2022.1052542 36466925 PMC9713810

[7] Kim ST Cristescu R Bass AJ Kim K-M Odegaard JI Kim K. Comprehensive molecular characterization of clinical responses to PD-1 inhibition in metastatic gastric cancer. Nat Med. (2018) 24:1449–58. doi: 10.1038/s41591-018-0101-z 30013197

[8] McGrail DJ Pilié PG Rashid NU Voorwerk L Slagter M Kok M. High tumor mutation burden fails to predict immune checkpoint blockade response across all cancer types. Ann Oncol. (2021) 32:661–72. doi: 10.1016/j.annonc.2021.02.006 PMC8053682 33736924

[9] Di Bartolomeo M Morano F Raimondi A Miceli R Corallo S Tamborini E. Prognostic and predictive value of microsatellite instability, inflammatory reaction and PD-L1 in gastric cancer patients treated with either adjuvant 5-FU/LV or sequential FOLFIRI followed by cisplatin and docetaxel: A translational analysis from the ITACA-S trial. Oncologist. (2020) 25:e460–8. doi: 10.1634/theoncologist.2019-0471 PMC7066701 32162808

[10] Luen SJ Salgado R Fox S Savas P Eng-Wong J Clark E. Tumour-infiltrating lymphocytes in advanced HER2-positive breast cancer treated with pertuzumab or placebo in addition to trastuzumab and docetaxel: a retrospective analysis of the CLEOPATRA study. Lancet Oncol. (2017) 18:52–62. doi: 10.1016/S1470-2045(16)30631-3 27964843 PMC5477653

[11] Peng H Wu X Liu S He M Xie C Zhong R. Multiplex immunofluorescence and single-cell transcriptomic profiling reveal the spatial cell interaction networks in the non-small cell lung cancer microenvironment. Clin Transl Med. (2023) 13:e1155. doi: 10.1002/ctm2.1155 36588094 PMC9806015

[12] Wu F Jiang T Chen G Huang Y Zhou J Lin L. Multiplexed imaging of tumor immune microenvironmental markers in locally advanced or metastatic non-small-cell lung cancer characterizes the features of response to PD-1 blockade plus chemotherapy. Cancer Commun (Lond). (2022) 42:1331–46. doi: 10.1002/cac2.12383 PMC9759770 36331328

[13] Kojima T Shah MA Muro K Francois E Adenis A Hsu C-H. Randomized phase III KEYNOTE-181 study of pembrolizumab versus chemotherapy in advanced esophageal cancer. J Clin Oncol. (2020) 38:4138–48. doi: 10.1200/JCO.20.01888 33026938

[14] Hu P Huang Q Li Z Wu X Ouyang Q Chen J. Silencing MAP3K1 expression through RNA interference enhances paclitaxel-induced cell cycle arrest in human breast cancer cells. Mol Biol Rep. (2014) 41:19–24. doi: 10.1007/s11033-013-2811-0 24253898

[15] Pérez-Ramírez C Cañadas-Garre M Alnatsha A Villar E Delgado JR Faus-Dáder MJ. Pharmacogenetic predictors of toxicity to platinum based chemotherapy in non-small cell lung cancer patients. Pharmacol Res. (2016) 111:877–84. doi: 10.1016/j.phrs.2016.08.002 27498158

[16] Shiotani B Zou L. Single-stranded DNA orchestrates an ATM-to-ATR switch at DNA breaks. Mol Cell. (2009) 33:547–58. doi: 10.1016/j.molcel.2009.01.024 PMC2675165 19285939

[17] Liu B Wang Y Wang H Li Z Yang L Yan S. RBM10 deficiency is associated with increased immune activity in lung adenocarcinoma. Front Oncol. (2021) 11:677826. doi: 10.3389/fonc.2021.677826 34367963 PMC8336464

[18] Zhang P Zhang H Tang J Ren Q Zhang J Chi H. The integrated single-cell analysis developed an immunogenic cell death signature to predict lung adenocarcinoma prognosis and immunotherapy. Aging (Albany NY). (2023) 15(19):10305–29. doi: 10.18632/aging.v15i19 PMC10599752 37796202

[19] Vihervuori H Autere TA Repo H Kurki S Kallio L Lintunen MM. Tumor-infiltrating lymphocytes and CD8+ T cells predict survival of triple-negative breast cancer. J Cancer Res Clin. (2019) 145:3105–14. doi: 10.1007/s00432-019-03036-5 PMC6861359 31562550

[20] Zhu Y Li M Mu D Kong L Zhang J Zhao F. CD8+/FOXP3+ ratio and PD-L1 expression associated with survival in pT3N0M0 stage esophageal squamous cell cancer. Oncotarget. (2016) 7:71455–65. doi: 10.18632/oncotarget.v7i44 PMC5342092 27683115

[21] Maibach F Sadozai H Seyed Jafari SM Hunger RE Schenk M. Tumor-infiltrating lymphocytes and their prognostic value in cutaneous melanoma. Front Immunol. (2020) 11:2105. doi: 10.3389/fimmu.2020.02105 33013886 PMC7511547

[22] Liang H Li H Xie Z Jin T Chen Y Lv Z. Quantitative multiplex immunofluorescence analysis identifies infiltrating PD1+ CD8+ and CD8+ T cells as predictive of response to neoadjuvant chemotherapy in breast cancer. Thorac Cancer. (2020) 11:2941–54. doi: 10.1111/1759-7714.13639 PMC7529566 32894006

[23] Chen Y Jia K Sun Y Zhang C Li Y Zhang L. Predicting response to immunotherapy in gastric cancer via multi-dimensional analyses of the tumour immune microenvironment. Nat Commun. (2022) 13:4851. doi: 10.1038/s41467-022-32570-z 35982052 PMC9388563

[24] Cabrita R Lauss M Sanna A Donia M Skaarup L Mathilde M. Tertiary lymphoid structures improve immunotherapy and survival in melanoma. Nature. (2020) 577:561–5. doi: 10.1038/s41586-019-1914-8 31942071

[25] Zhang P Dong S Sun W Zhong W Xiong J Gong X. Deciphering Treg cell roles in esophageal squamous cell carcinoma: a comprehensive prognostic and immunotherapeutic analysis. Front Mol Biosci. (2023) 10. doi: 10.3389/fmolb.2023.1277530 PMC10568469 37842637

[26] Śledzińska A Vila de Mucha M Bergerhoff K Hotblack A Demane DF Ghorani E. Regulatory T cells restrain interleukin-2- and blimp-1-dependent acquisition of cytotoxic function by CD4+ T cells. Immunity. (2020) 52:151–166.e156. doi: 10.1016/j.immuni.2019.12.007 31924474 PMC7369640

[27] Lhuillier C Rudqvist NP Yamazaki T Zhang T Charpentier M Galluzzi L. Radiotherapy-exposed CD8+ and CD4+ neoantigens enhance tumor control. J Clin Invest. (2021) 131(5). doi: 10.1172/JCI138740 PMC7919731 33476307

[28] Kurachi M. CD8+ T cell exhaustion. Semin Immunopathol. (2019) 41:327–37. doi: 10.1007/s00281-019-00744-5 30989321

[29] Cai L Li Y Tan J Xu L Li Y. Targeting LAG-3, TIM-3. J Hematol Oncol. (2023) 16:101. doi: 10.1186/s13045-023-01499-1 37670328 PMC10478462

[30] Tan J Yu Z Huang J Chen Y Huang S Yao D. Increased PD-1+Tim-3+ exhausted T cells in bone marrow may influence the clinical outcome of patients with AML. biomark Res. (2020) 8:6. doi: 10.1186/s40364-020-0185-8 32082573 PMC7020501

[31] Ausejo-Mauleon I Labiano S de la Nava DP Laspidea V Zalacain M Marrodán L. TIM-3 blockade in diffuse intrinsic pontine glioma models promotes tumor regression and antitumor immune memory. ancer Cell. (2023) 41:1911–1926.e8. doi: 10.1016/j.ccell.2023.09.001 PMC10644900 37802053

[32] Rezaei M Tan J Zeng C Li Y Ganjalikhani-Hakemi M. TIM - 3 in leukemia; immune response and beyond. Front Oncol. (2021) 11:753677. doi: 10.3389/fonc.2021.753677 34660319 PMC8514831

[33] Tan J Tan H Li Y. Targeting TIM-3 for hematological Malignancy: latest updates from the 2022 ASH annual meeting. Exp Hematol Oncol. (2023) 12:62. doi: 10.1186/s40164-023-00421-2 37468979 PMC10357734

[34] Bailly C Thuru X Goossens L Goossens J-F. Soluble TIM-3 as a biomarker of progression and therapeutic response in cancers and other of human diseases. Biochem Pharmacol. (2023) 209:115445. doi: 10.1016/j.bcp.2023.115445 36739094

[35] Sauer N Janicka N Szlasa W Skinderowicz B Kołodzińska K Dwernicka W. TIM-3 as a promising target for cancer immunotherapy in a wide range of tumors. Cancer Immunol Immun. (2023) 72:3405–25. doi: 10.1007/s00262-023-03516-1 PMC10576709 37567938

[36] Kato R Yamasaki M Urakawa S Nishida K Makino T Morimoto-Okazawa A. Increased Tim-3+ T cells in PBMCs during nivolumab therapy correlate with responses and prognosis of advanced esophageal squamous cell carcinoma patients. Cancer Immunol Immun. (2018) 67:1673–83. doi: 10.1007/s00262-018-2225-x PMC11028199 30128737

[37] Qian D Wang Y Zhao G Cao F Er P Chen X. Tumor remission and tumor-infiltrating lymphocytes during chemoradiation therapy: predictive and prognostic markers in locally advanced esophageal squamous cell carcinoma. Int J Radiat Oncol. (2019) 105:319–28. doi: 10.1016/j.ijrobp.2019.06.079 31228553

[38] Han R Zhang Y Wang T Xiao H Luo Z Shen C. Tumor immune microenvironment predicts the pathologic response of neoadjuvant chemoimmunotherapy in non-small-cell lung cancer. Cancer Sci. (2023) 114:2569–83. doi: 10.1111/cas.15778 PMC10236616 36880750

[39] Wang S Yuan P Mao B Li N Ying J Tao X. Genomic features and tumor immune microenvironment alteration in NSCLC treated with neoadjuvant PD-1 blockade. NPJ Precis Oncol. (2022) 6:2. doi: 10.1038/s41698-021-00244-6 35027673 PMC8758728

[40] Zhou YH Li JY Yan JX Guo P He W-W Liu YA. Alleviation of neoadjuvant immunochemotherapy for esophageal squamous cell carcinoma and its relationship with expression and changes of PD-L1. Neoplasma. (2022) 69:785–93. doi: 10.4149/neo_2022_211223N1826 35400168

[41] Chen Y Ren M Li B Meng Y Wang C Jiang P. Neoadjuvant sintilimab plus chemotherapy for locally advanced resectable esophageal squamous cell carcinoma: a prospective, single-arm, phase II clinical trial (CY-NICE). J Thorac Dis. (2023) 15:6761–75. doi: 10.21037/jtd-23-1388 PMC10797361 38249875

[42] Tian Y Shi Z Wang C Ke S Qiu H Zhao W. A comparison of clinicopathologic outcomes and patterns of lymphatic spread across neoadjuvant chemotherapy, neoadjuvant chemoradiotherapy, and neoadjuvant immunochemotherapy in locally advanced esophageal squamous cell carcinoma. Ann Surg Oncol. (2023) 31:860–71. doi: 10.1245/s10434-023-14534-9 37947979

[43] Chen P Wang L Yang X Feng J. Lymph node ratio is a prognostic indicator for locally advanced esophageal squamous cell carcinoma after neoadjuvant immunochemotherapy. Biomolecules & Biomedicine. (2024) 24(1):159–69. doi: 10.17305/bb.2023.9435 PMC10787609 37597214

[44] Fu C Feng S Wang S Su X. Development and validation of a prognostic model for esophageal carcinoma based on immune microenvironment using system bioinformatics. Cancer Med. (2022) 12:2089–103. doi: 10.1002/cam4.4985 PMC9883539 35771026

[45] Feng J Wang L Yang X Chen Q Cheng X. Pretreatment pan-immune-inflammation value (PIV) in predicting therapeutic response and clinical outcomes of neoadjuvant immunochemotherapy for esophageal squamous cell carcinoma. Ann Surg Oncol. (2023) 31:272–83. doi: 10.1245/s10434-023-14430-2 37838648

[46] Feng J Wang L Yang X Chen Q Cheng X. A novel immune-nutritional score predicts response to neoadjuvant immunochemotherapy after minimally invasive esophagectomy for esophageal squamous cell carcinoma. Front Immunol. (2023) 14:1217967. doi: 10.3389/fimmu.2023.1217967 37954582 PMC10634314

