# Supplementary figures and images for: To develop a prognostic model for neoadjuvant immunochemotherapy efficacy in esophageal squamous cell carcinoma by analyzing the immune microenvironment
Source: Front Immunol. 2024 Apr 25;15:1312380. doi: 10.3389/fimmu.2024.1312380 (PMC11079241; doi:10.3389/fimmu.2024.1312380)

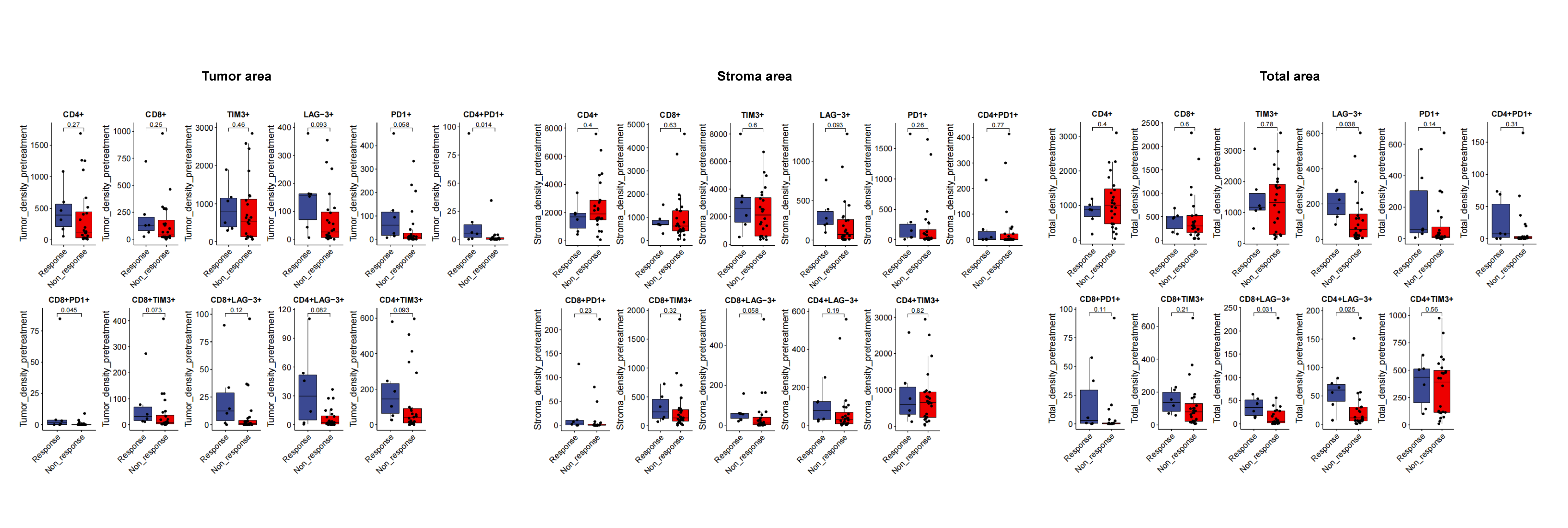

Supplement: Supplementary Figure 1 — Quantitative analysis results of panel 1. [file Image_1.tif]

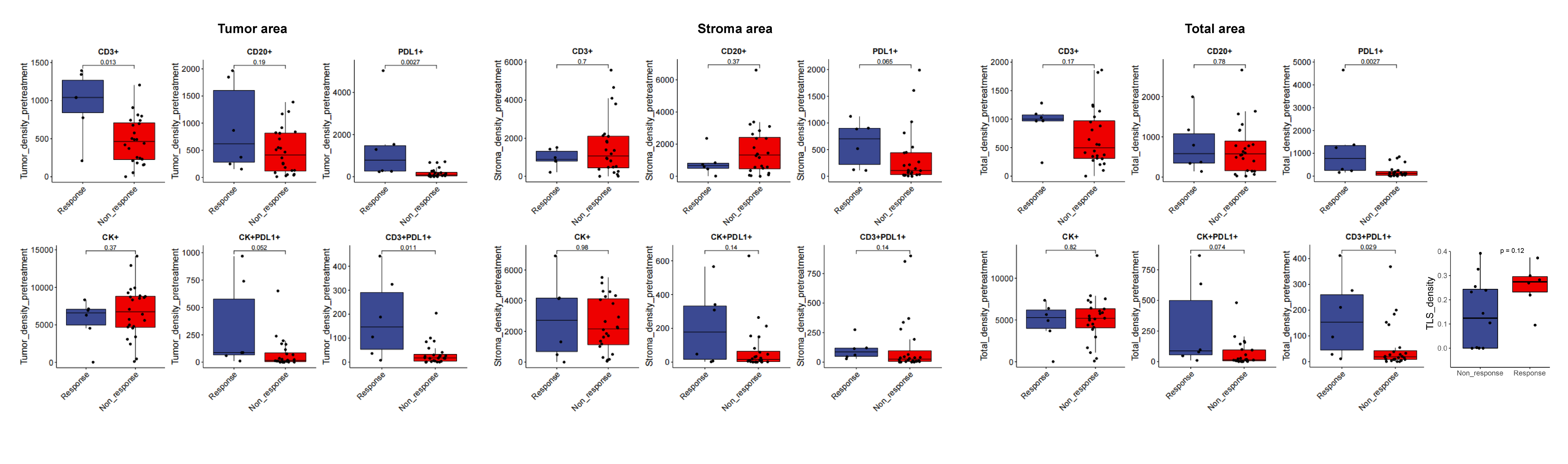

Supplement: Supplementary Figure 2 — Quantitative analysis results of panel 2. [file Image_2.tif]

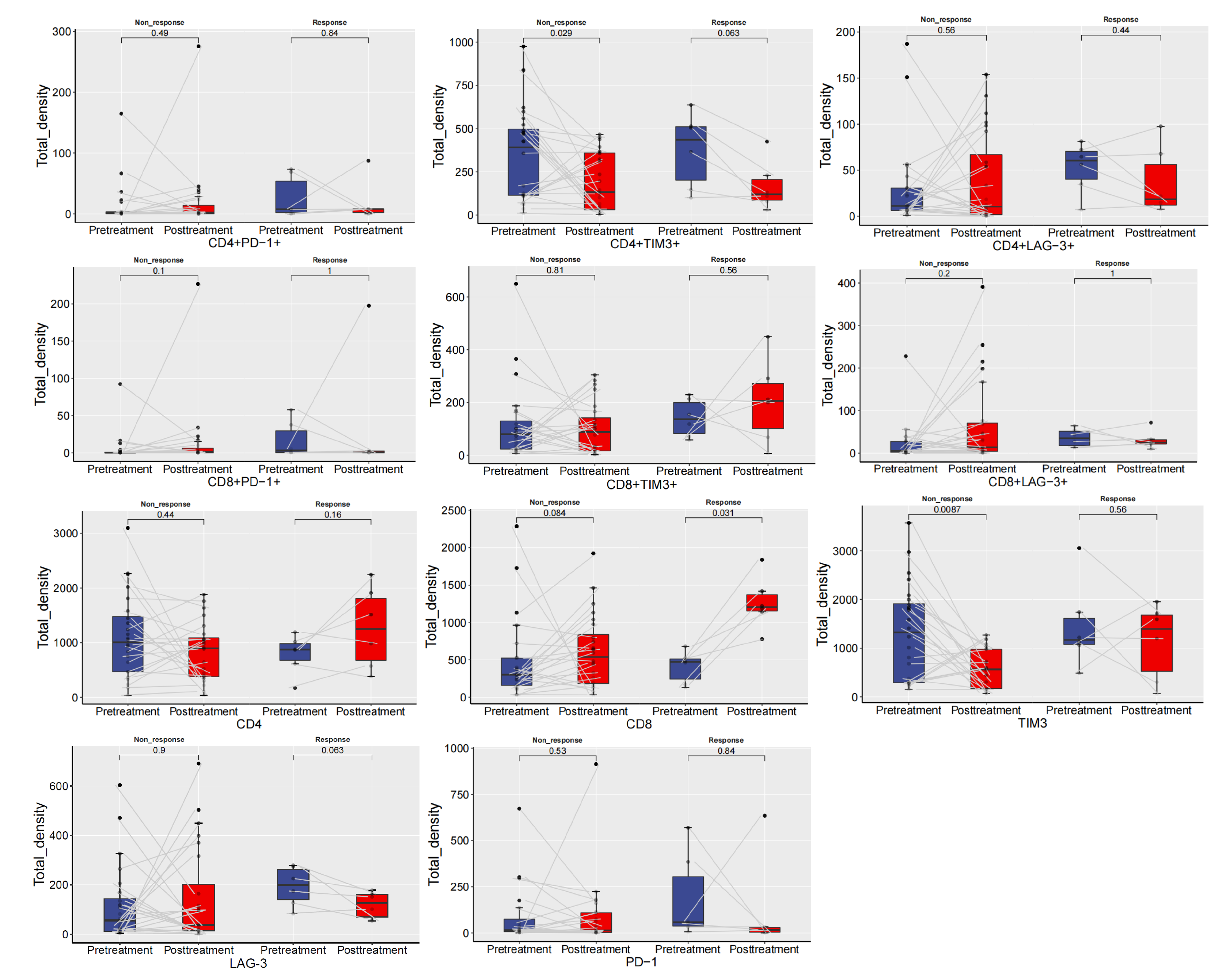

Supplement: Supplementary Figure 3 — The dynamic analysis results of the two groups before and after treatment. [file Image_3.tif]

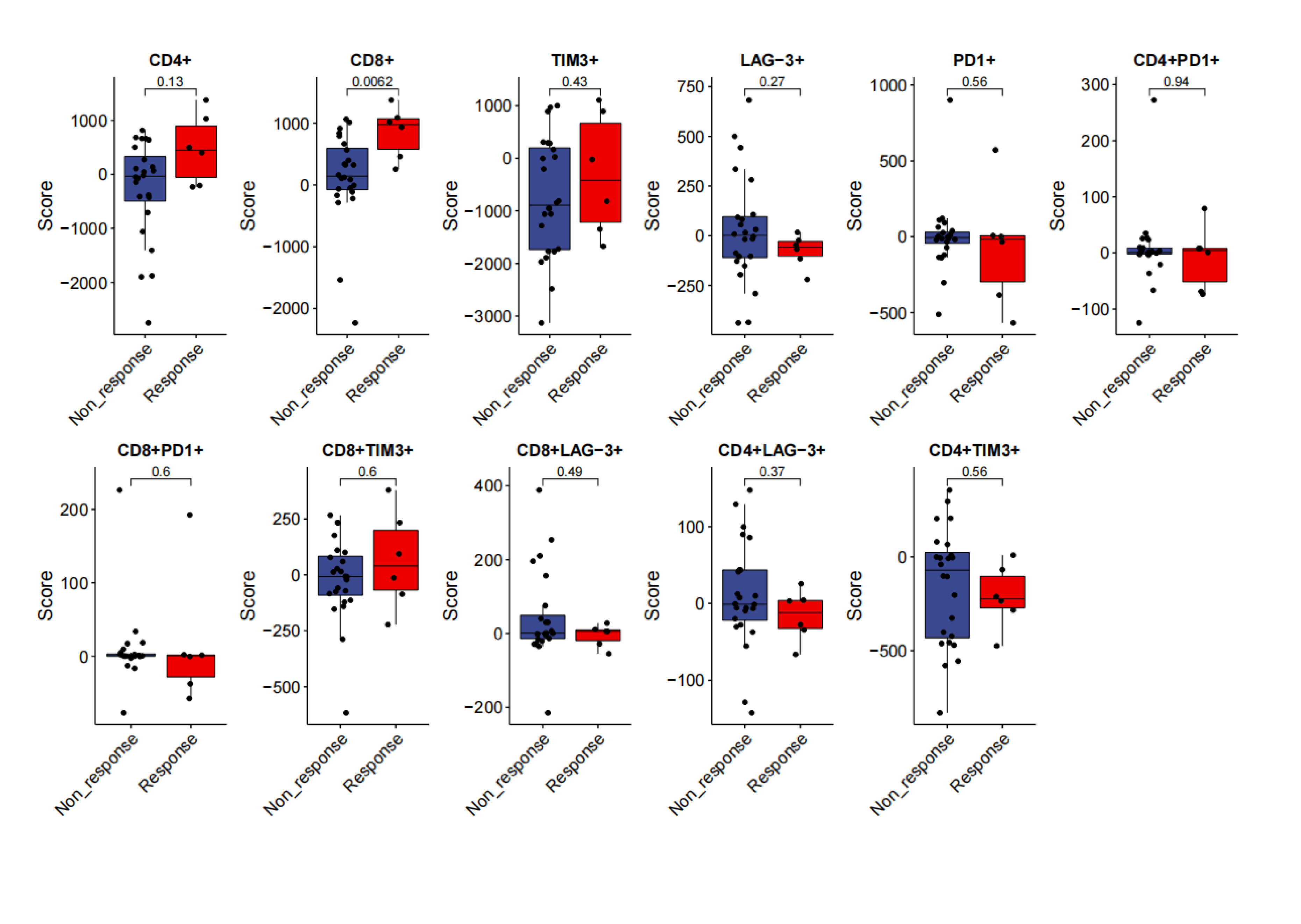

Supplement: Supplementary Figure 4 — The variation value analysis results of the two groups before and after treatment. [file Image_4.tif]

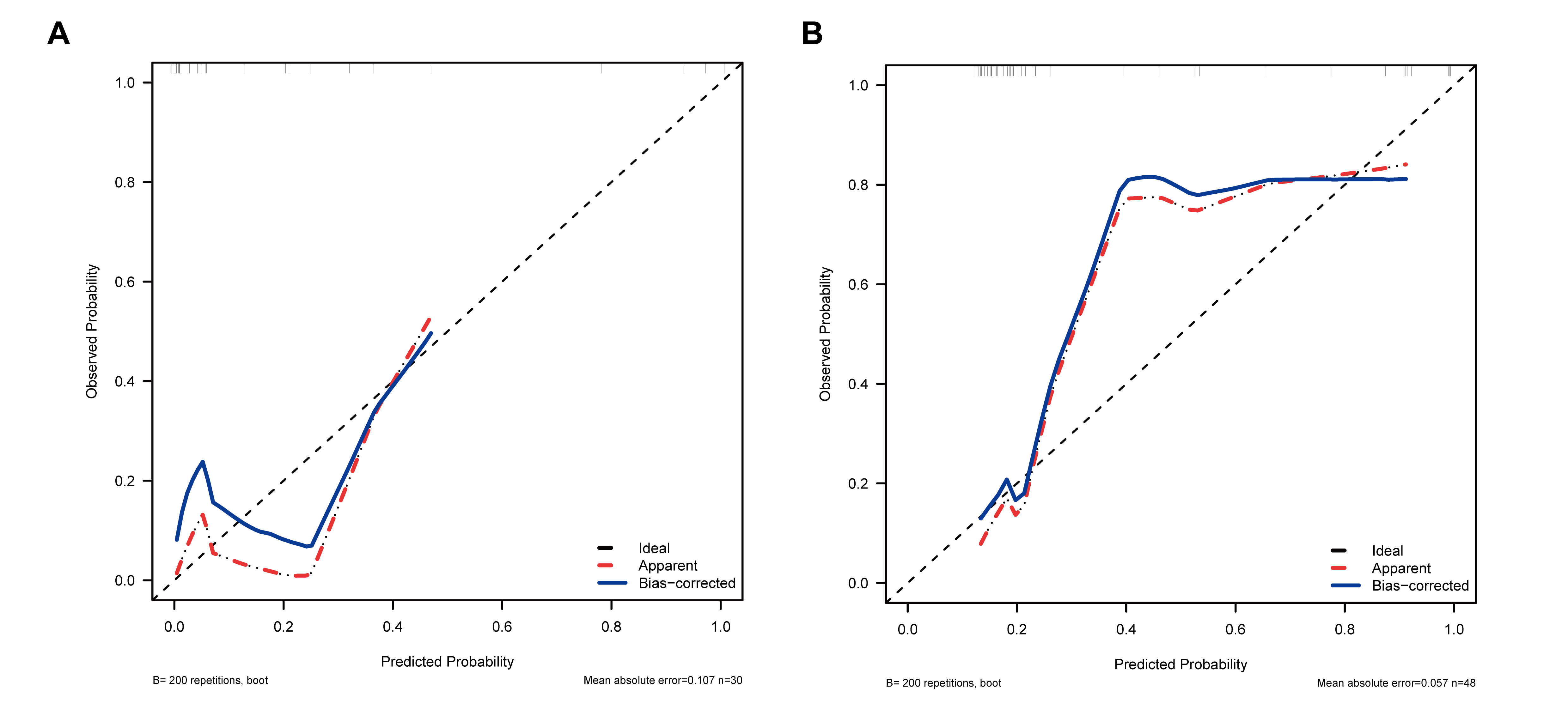

Supplement: Supplementary Figure 5 — Model calibration curves in training set (A) and validation set (B). [file Image_5.tif]

SNV Heatmap

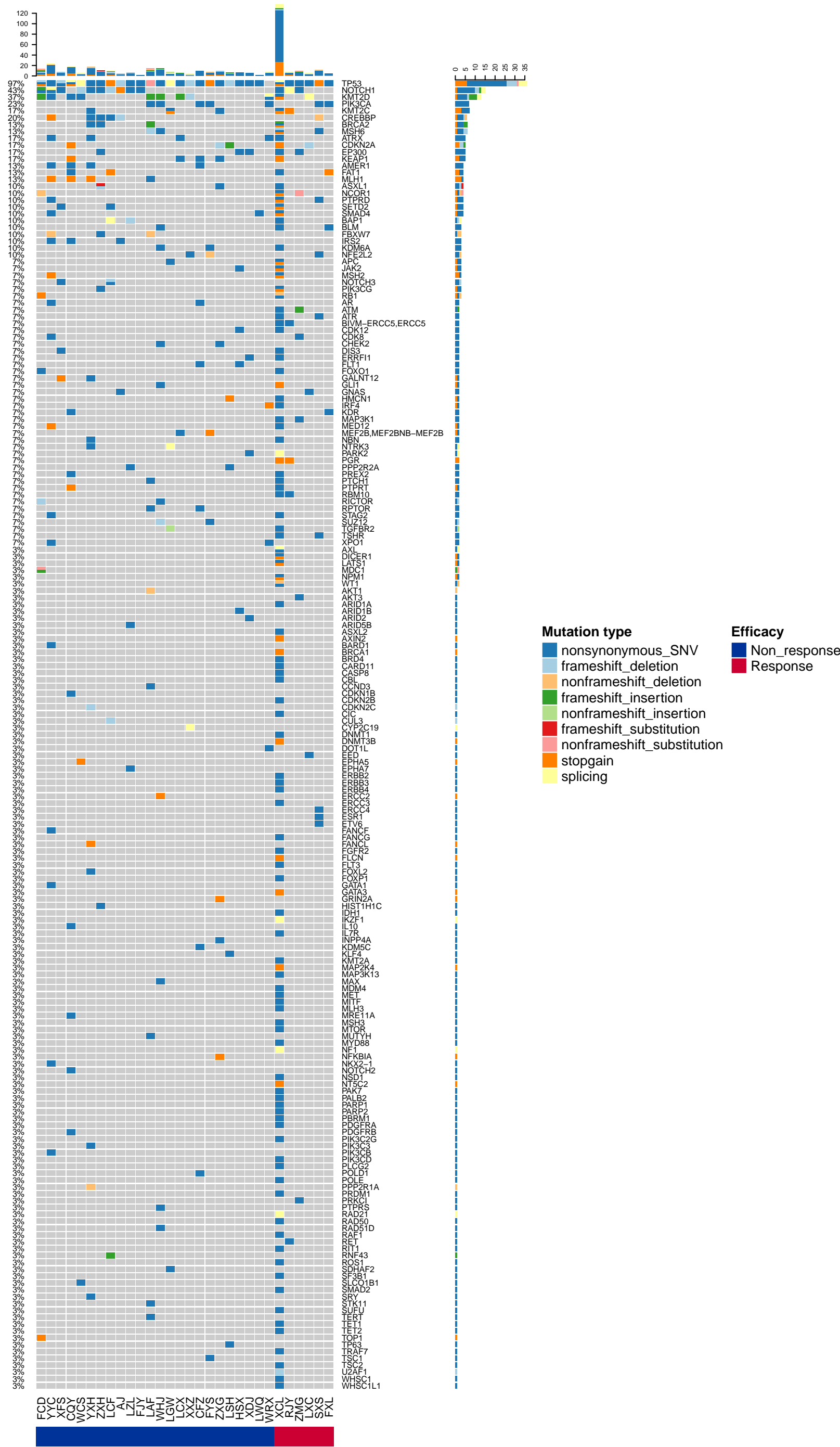

Supplement: Supplementary Table 1 — Clinicopathological characteristics of validation set. [file DataSheet_1.pdf]

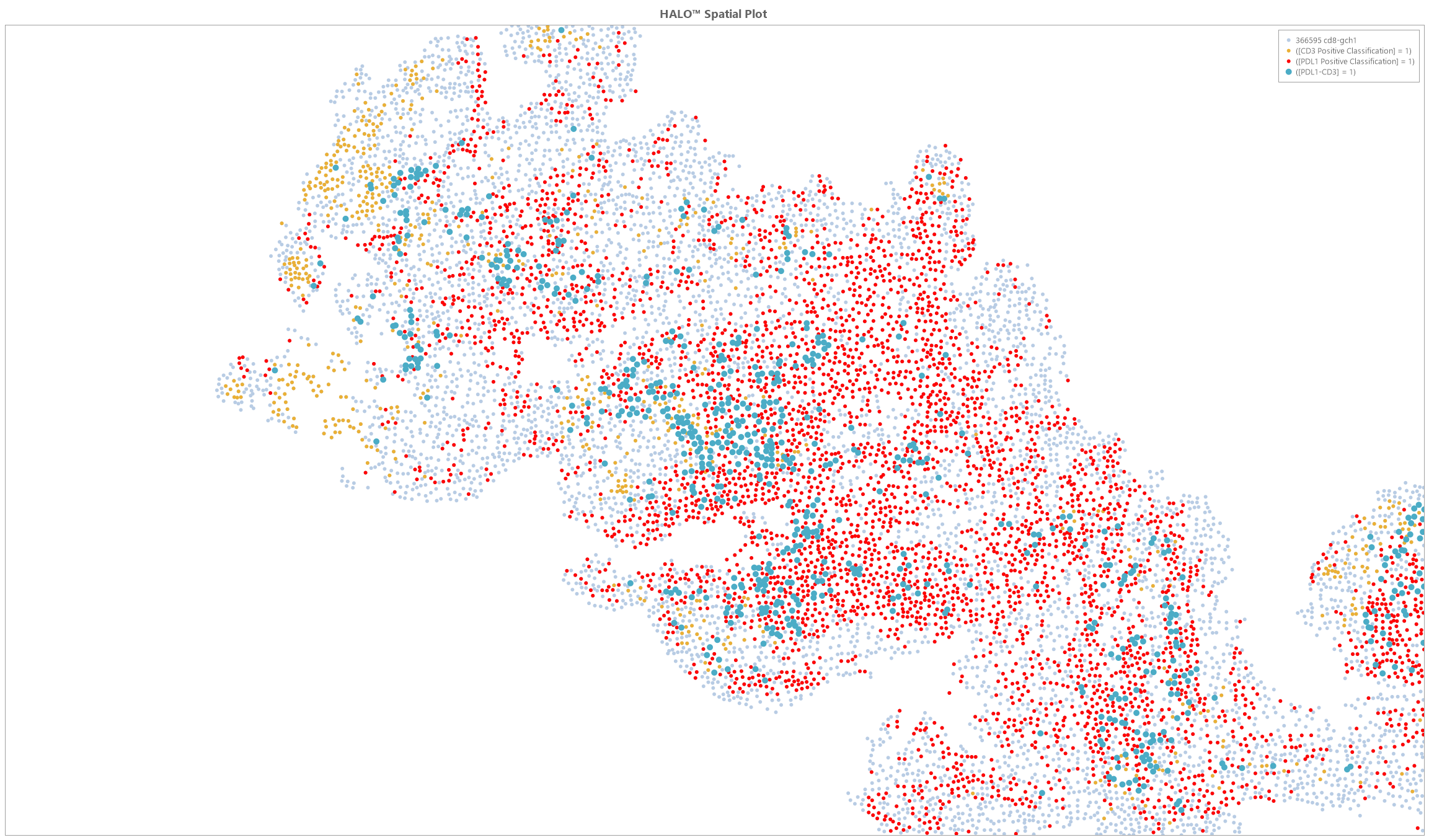

Supplement: Supplementary file 8 [file DataSheet_2.zip › AI-PDL1-CD3-1.png]

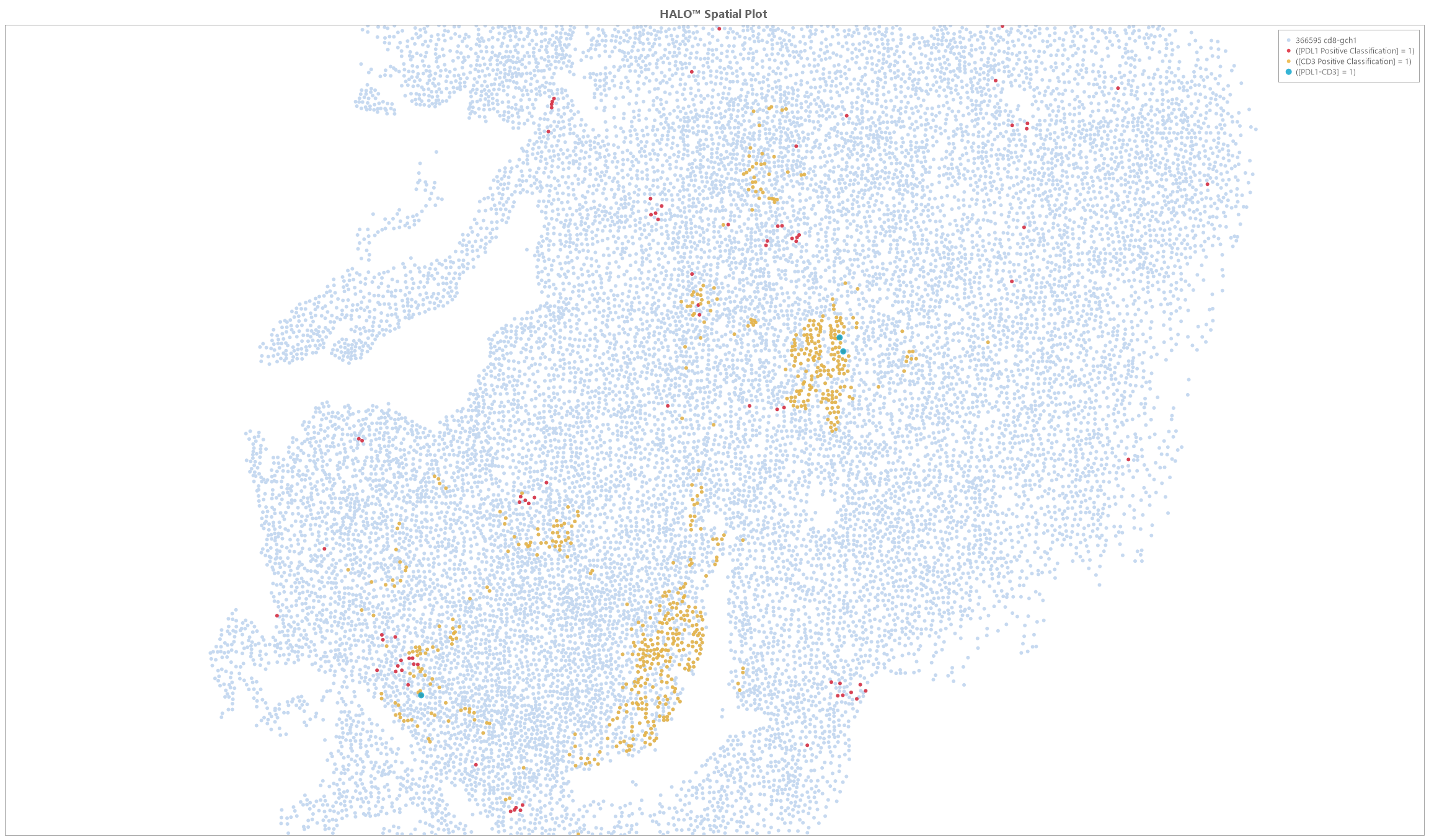

Supplement: Supplementary file 8 [file DataSheet_2.zip › AI-PDL1-CD3-2.png]

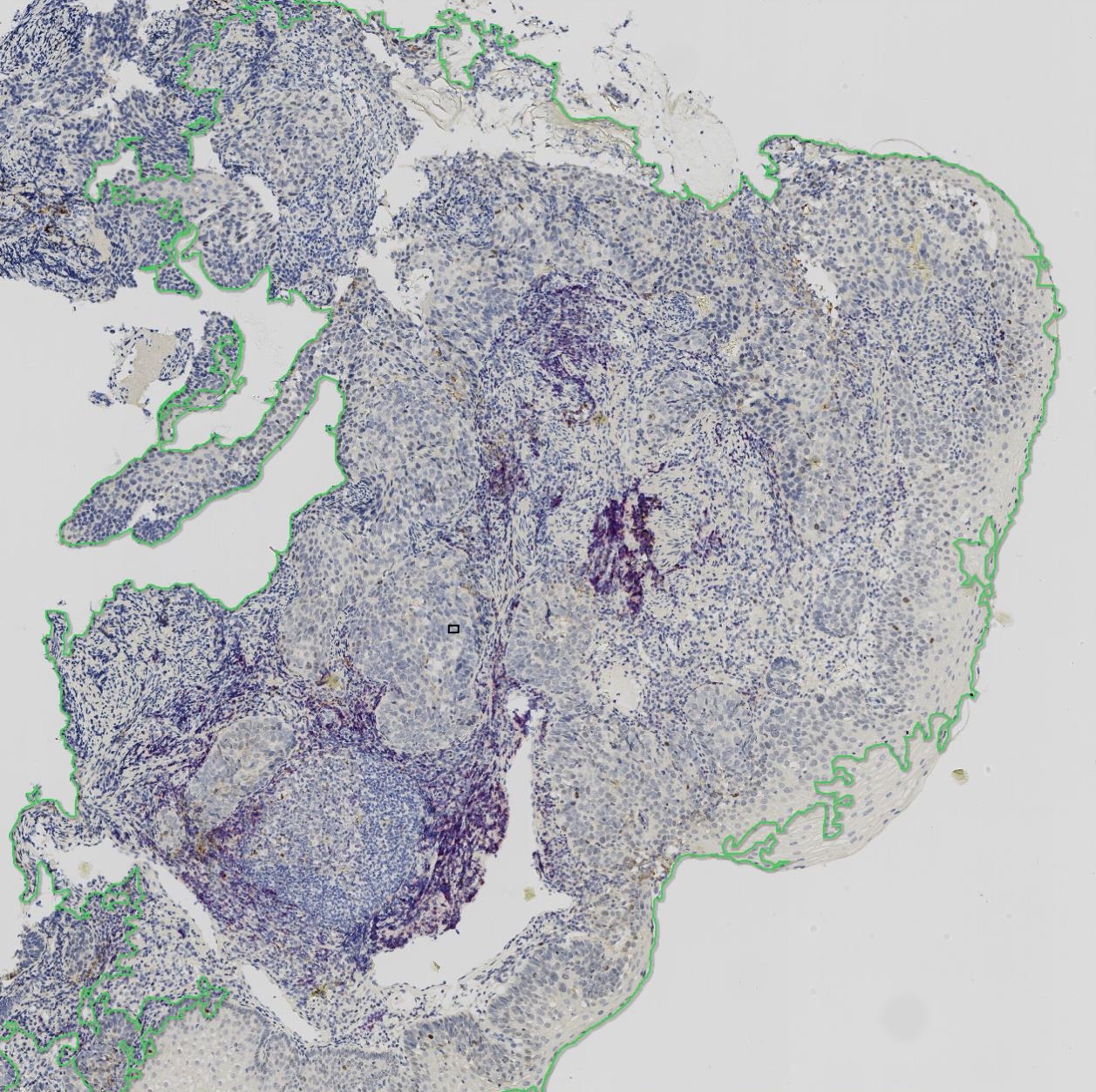

Supplement: Supplementary file 8 [file DataSheet_2.zip › Fig7A-IHC-Nonres.png]

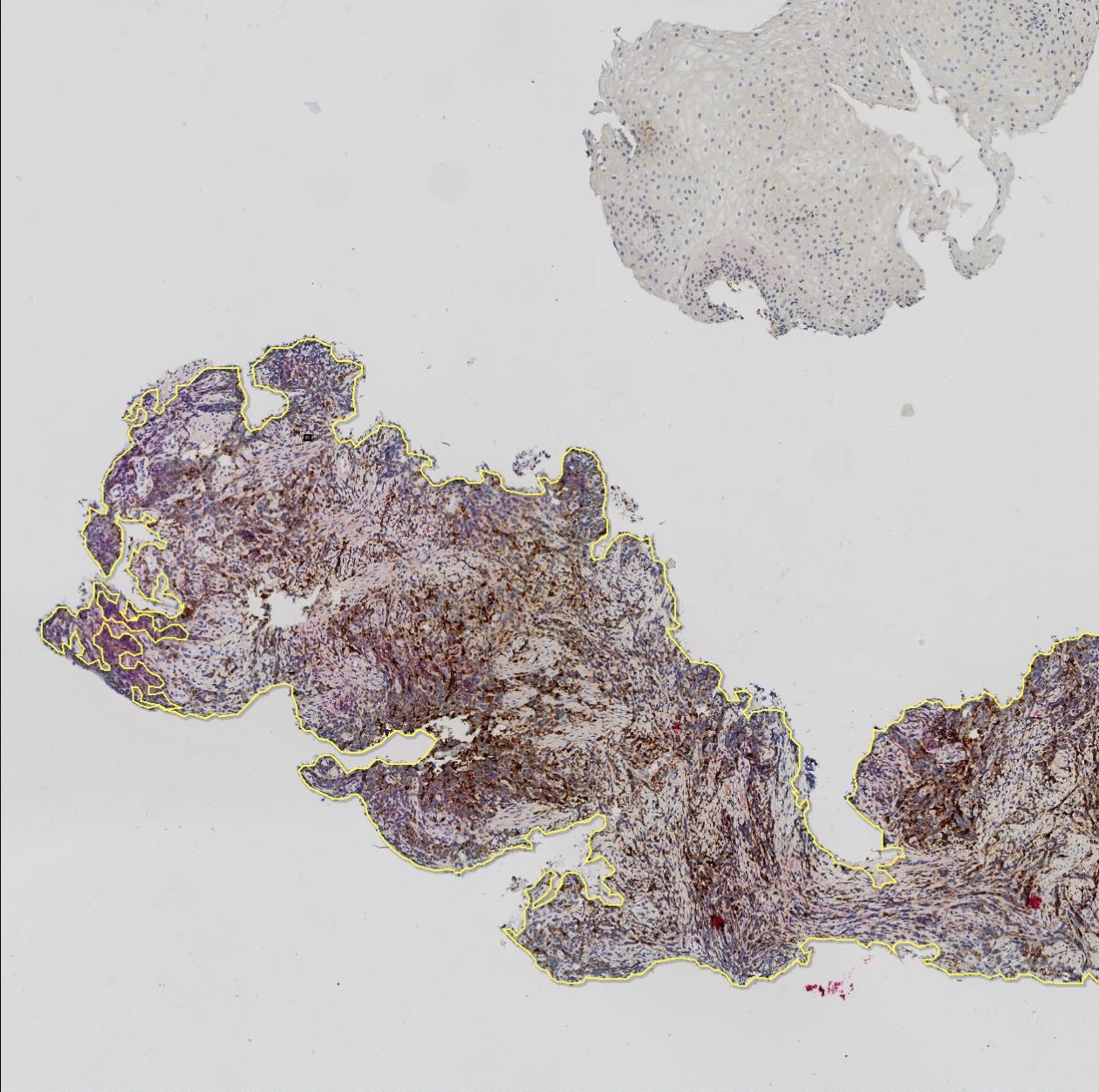

Supplement: Supplementary file 8 [file DataSheet_2.zip › Fig7A-IHC-Res.png]

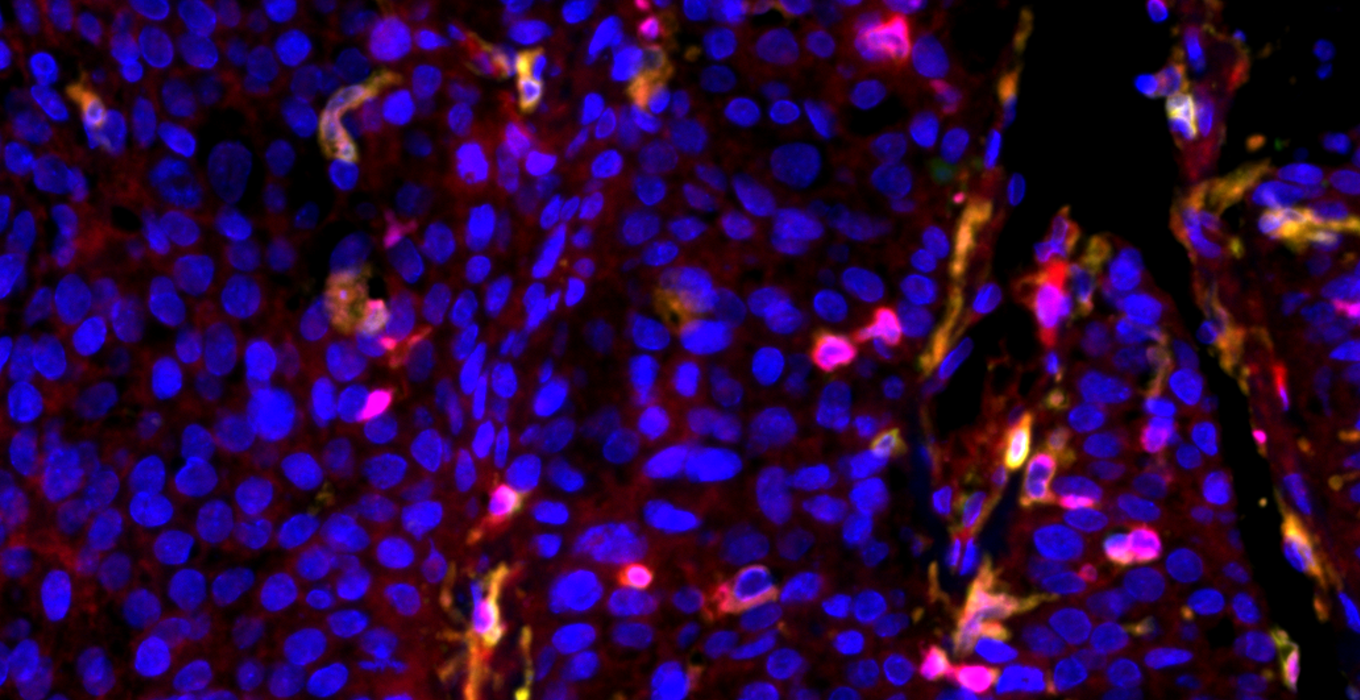

Supplement: Supplementary file 8 [file DataSheet_2.zip › Slide MIF-M1-P1-Nonresponse-High power field.BMP]

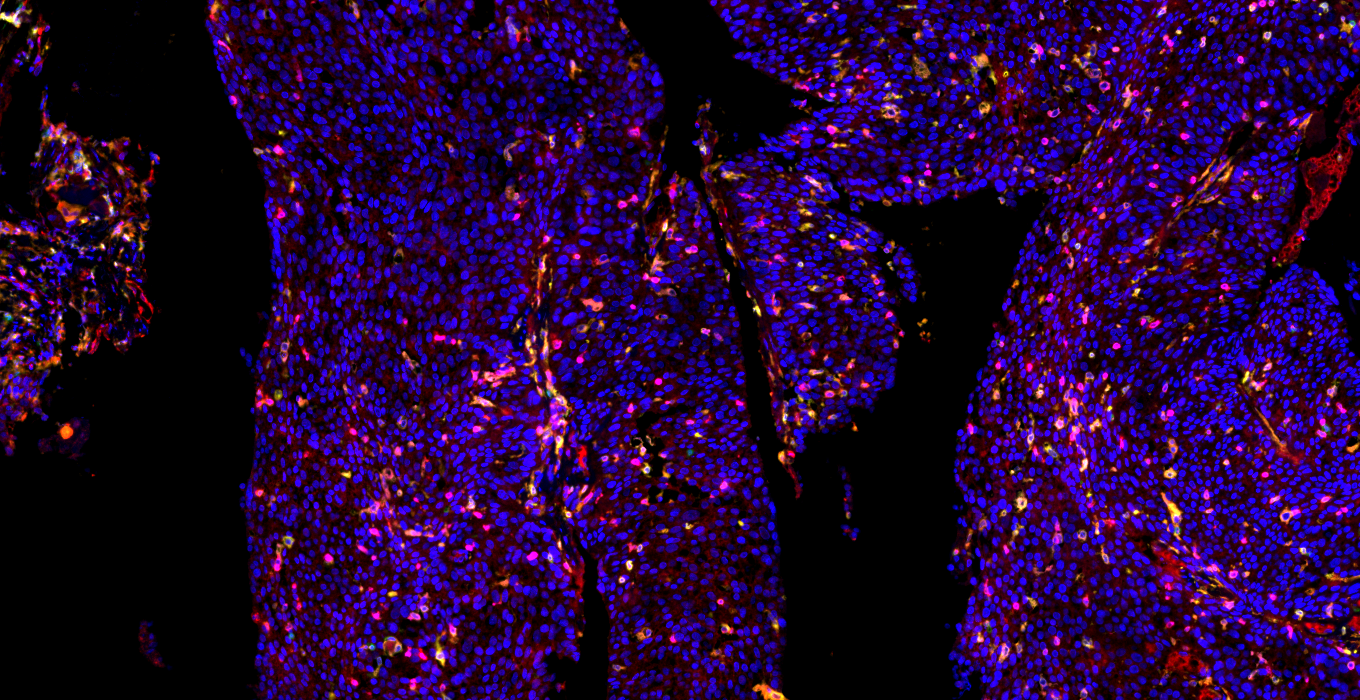

Supplement: Supplementary file 8 [file DataSheet_2.zip › Slide MIF-M1-P1-Nonresponse-Low power field.BMP]

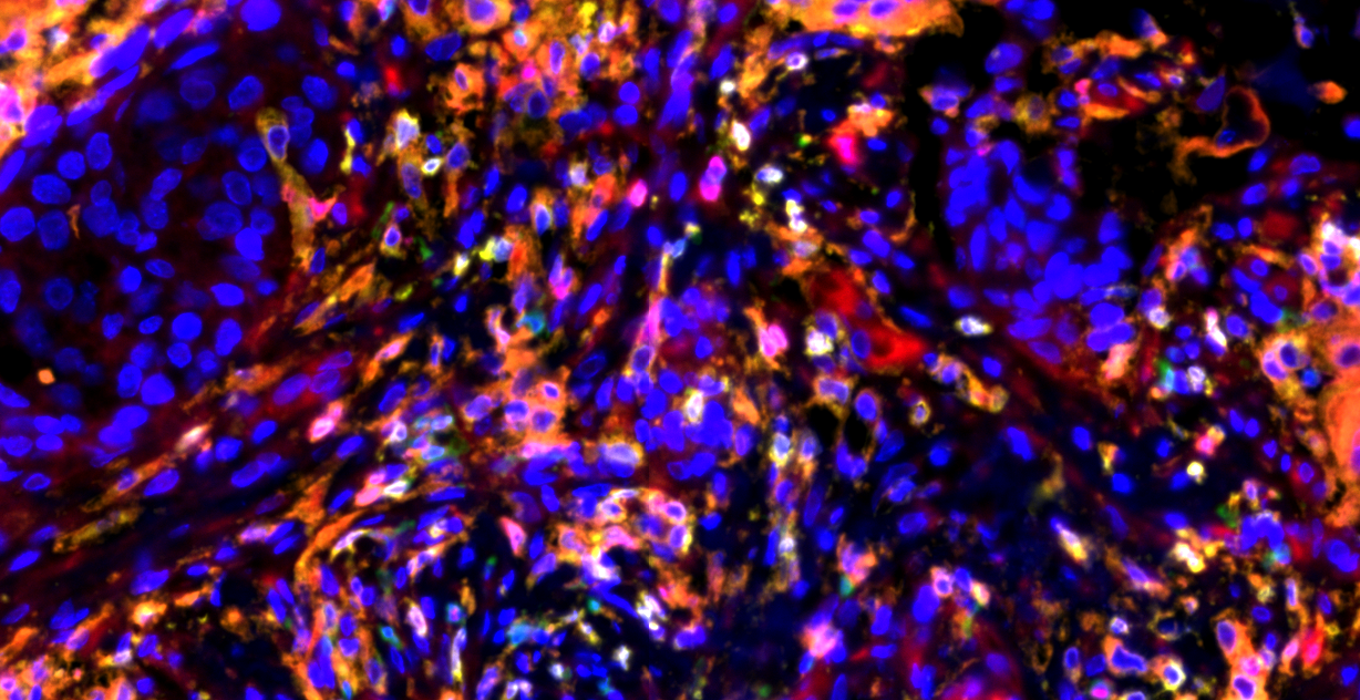

Supplement: Supplementary file 8 [file DataSheet_2.zip › Slide MIF-M1-P1-Response-High power field.BMP]

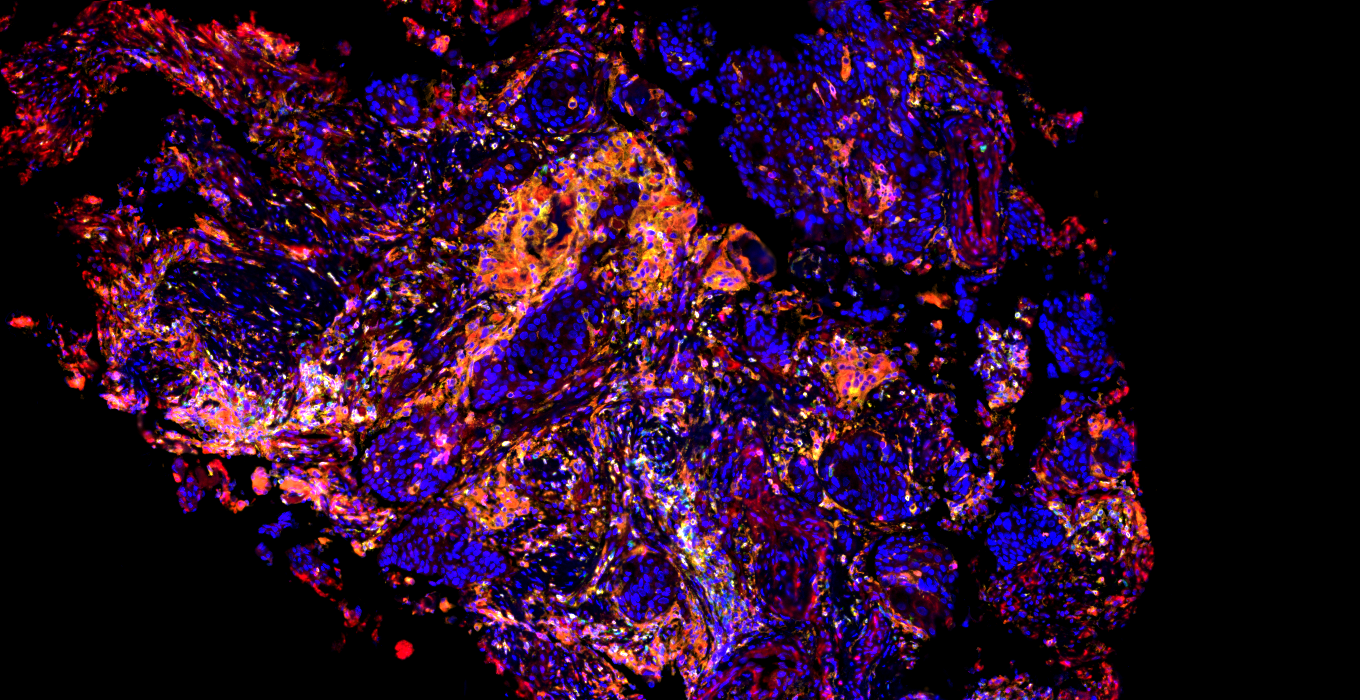

Supplement: Supplementary file 8 [file DataSheet_2.zip › Slide MIF-M1-P1-Response-Low power field.BMP]

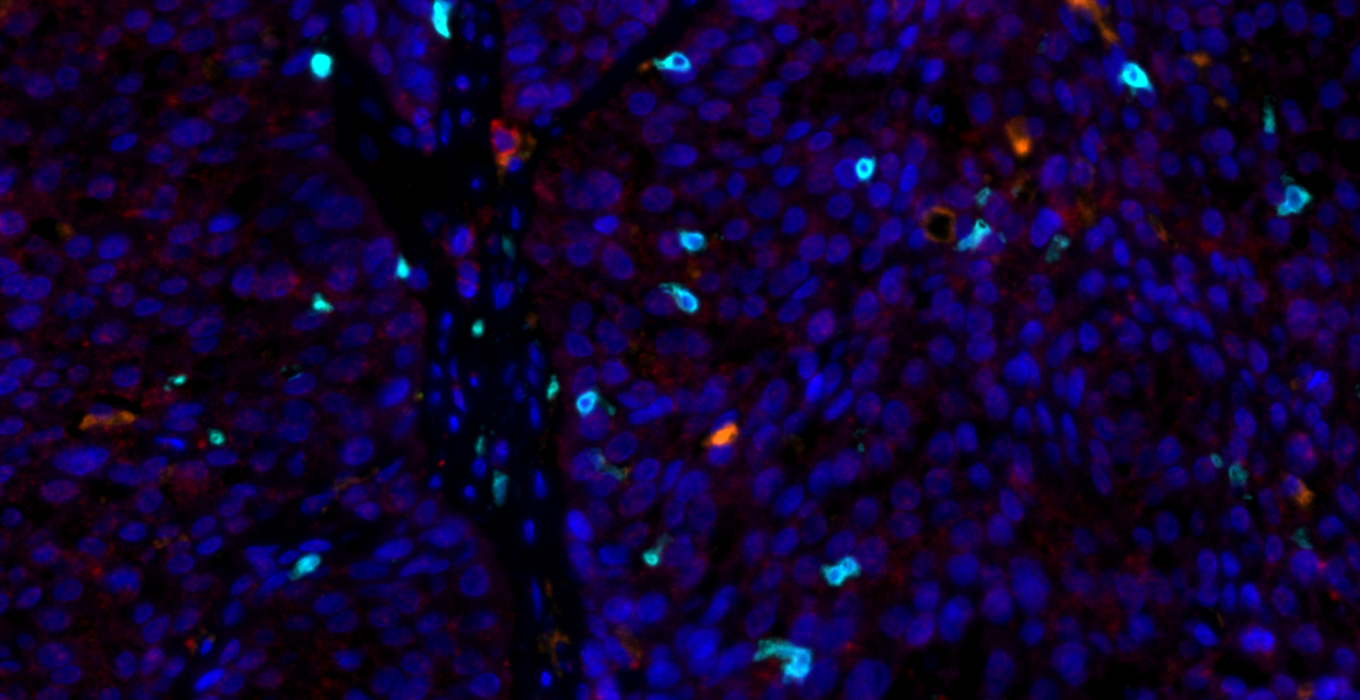

Supplement: Supplementary file 8 [file DataSheet_2.zip › Slide MIF-M2-P2-Nonresponse-High power field.BMP]

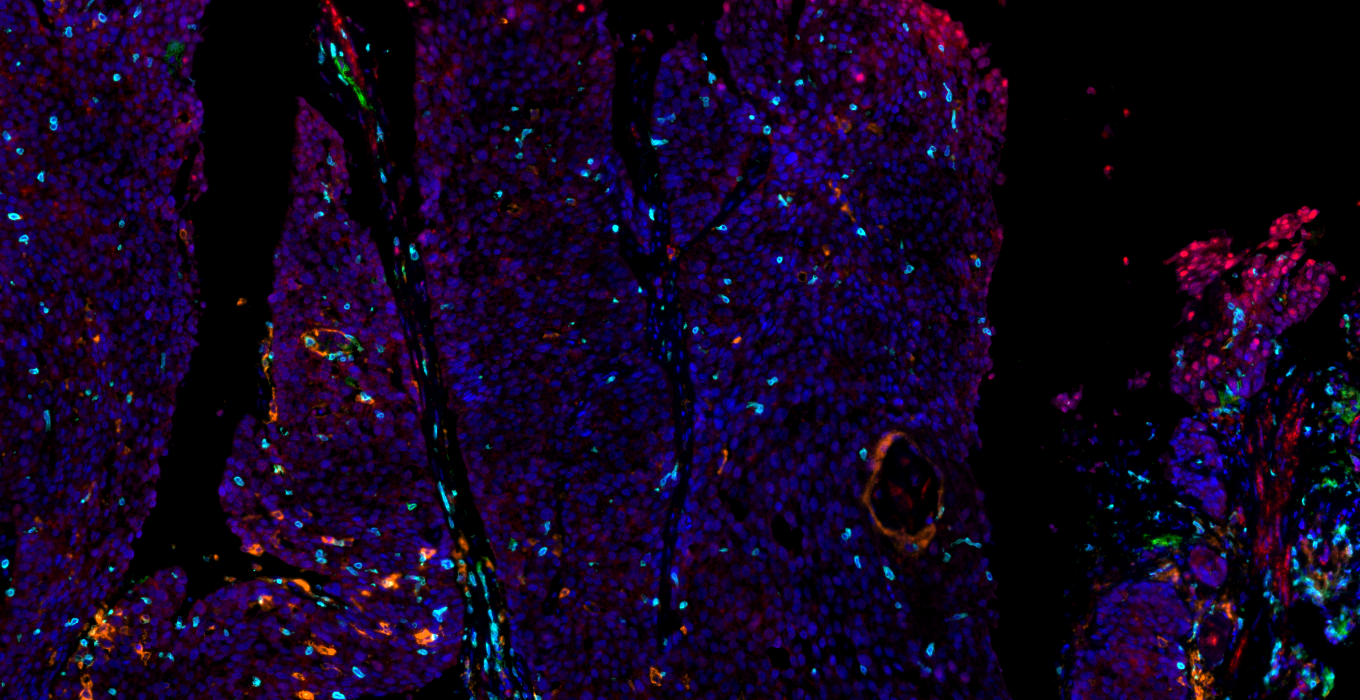

Supplement: Supplementary file 8 [file DataSheet_2.zip › Slide MIF-M2-P2-Nonresponse-Low power field.BMP]

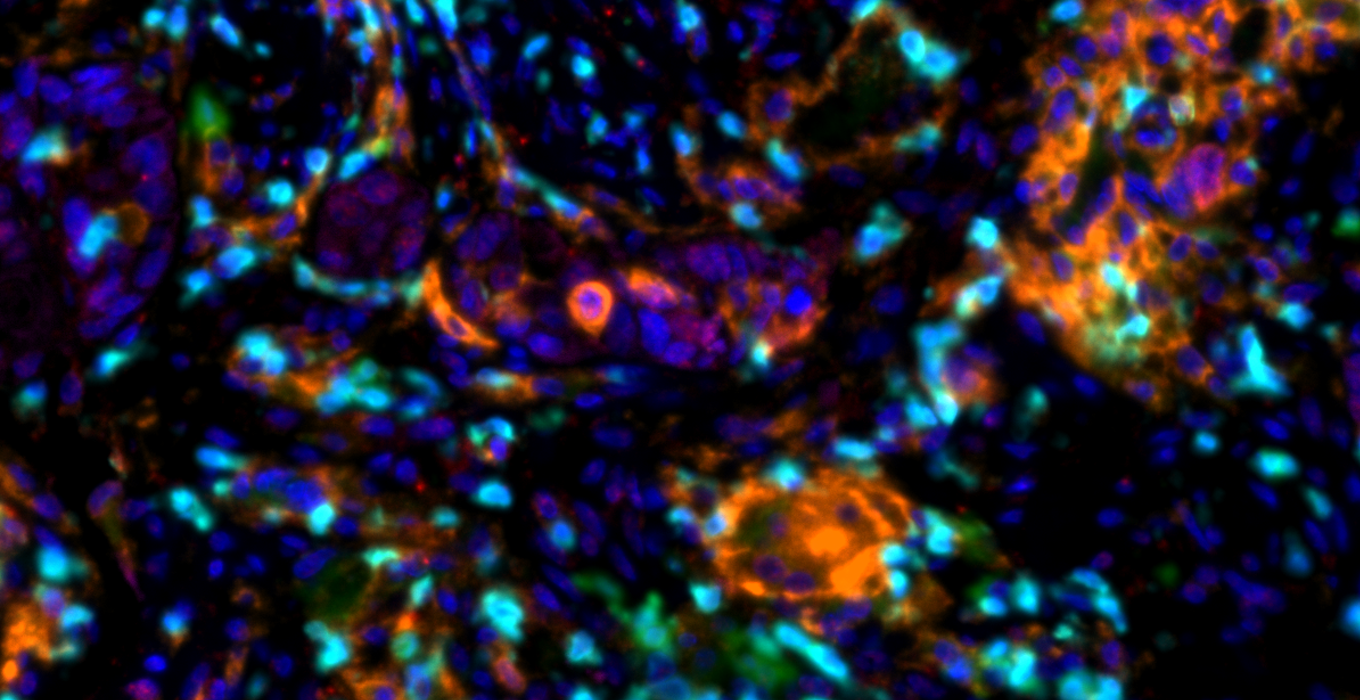

Supplement: Supplementary file 8 [file DataSheet_2.zip › Slide MIF-M2-P2-Response-High power field.BMP]

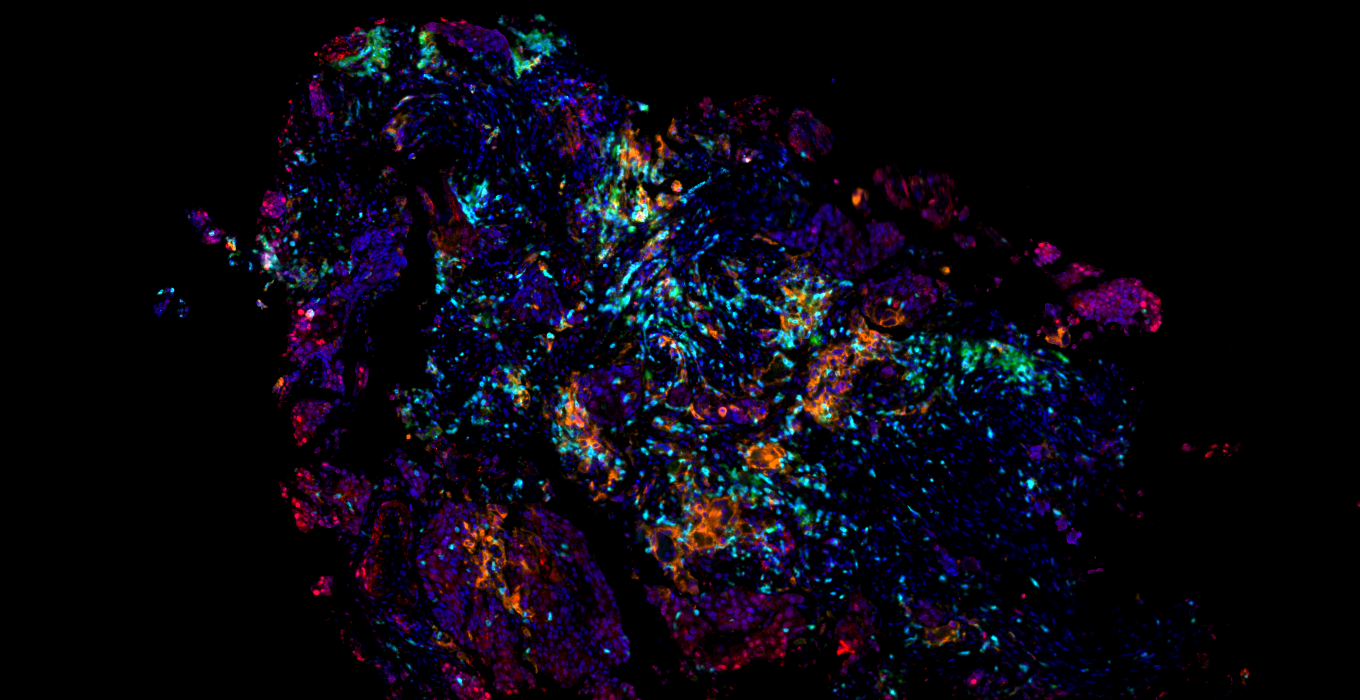

Supplement: Supplementary file 8 [file DataSheet_2.zip › Slide MIF-M2-P2-Response-Low power field.BMP]
