# Supplementary material for: To develop a prognostic model for neoadjuvant immunochemotherapy efficacy in esophageal squamous cell carcinoma by analyzing the immune microenvironment
Source: Front Immunol. 2024 Apr 25;15:1312380. doi: 10.3389/fimmu.2024.1312380 (PMC11079241; doi:10.3389/fimmu.2024.1312380)
Supplement: Supplementary Figure 6 — Patient mutation information. [file Table_1.docx]

**Supplementary Table.1 Clinicopathological Characteristics of Validation set**

| Characteristic | Total (n = 48) | Non-response  (n = 32) | Response  (n = 16) | p |
| --- | --- | --- | --- | --- |
| **Gender, n (%)** |  |  |  | 1 |
| **Female** | 7 (14.6) | 5 (15.6) | 2 (12.5) |  |
| **Male** | 41 (85.4) | 27 (84.4) | 14 (87.5) |  |
| **Age, n (%)** |  |  |  | 1 |
| **≤65** | 36 (75.0) | 24 (75) | 12 (75) |  |
| **＞65** | 12 (25.0) | 8 (25) | 4 (25) |  |
| **Cigarette smoking history, n (%)** |  |  |  | 0.286 |
| **No** | 17 (35.4) | 13 (40.6) | 4 (25) |  |
| **Yes** | 31 (64.6) | 19 (59.4) | 12 (75) |  |
| **Alcohol drinking history, n (%)** |  |  |  | 0.019 |
| **No** | 17 (35.4) | 15 (46.9) | 2 (12.5) |  |
| **Yes** | 31 (64.6) | 17 (53.1) | 14 (87.5) |  |
| **Family history, n (%)** |  |  |  | 0.144 |
| **No** | 37 (77.1) | 27 (84.4) | 10 (62.5) |  |
| **Yes** | 11 (22.9) | 5 (15.6) | 6 (37.5) |  |
| **cT, n (%)** |  |  |  | 1 |
| **T2** | 5 (10.4) | 3 (9.4) | 2 (12.5) |  |
| **T3** | 33 (68.8) | 22 (68.8) | 11 (68.8) |  |
| **T4** | 10 (20.8) | 7 (21.9) | 3 (18.8) |  |
| **cN, n (%)** |  |  |  | 0.032 |
| **0** | 4 ( 8.3) | 4 (12.5) | 0 (0) |  |
| **1** | 31 (64.6) | 23 (71.9) | 8 (50) |  |
| **2** | 13 (27.1) | 5 (15.6) | 8 (50) |  |
| **stage, n (%)** |  |  |  | 0.365 |
| **II** | 4 ( 8.3) | 4 (12.5) | 0 (0) |  |
| **III** | 34 (70.8) | 21 (65.6) | 13 (81.2) |  |
| **IV** | 10 (20.8) | 7 (21.9) | 3 (18.8) |  |
| **Differentiation, n (%)** |  |  |  | 0.758 |
| **Well** | 9 (18.8) | 6 (18.8) | 3 (18.8) |  |
| **Middle** | 30 (62.5) | 21 (65.6) | 9 (56.2) |  |
| **Poor** | 9 (18.8) | 5 (15.6) | 4 (25) |  |
| **Primary tumor location, n (%)** |  |  |  | 0.303 |
| **Lower** | 29 (60.4) | 17 (53.1) | 12 (75) |  |
| **Middle** | 15 (31.2) | 11 (34.4) | 4 (25) |  |
| **Upper** | 4 ( 8.3) | 4 (12.5) | 0 (0) |  |
